# Supplementary material for: Development of a mechatronic weft selector to enhance patterning capacity in Rapier looms
Source: PLoS One. 2025 Dec 9;20(12):e0338603. doi: 10.1371/journal.pone.0338603 (PMC12688087; doi:10.1371/journal.pone.0338603)
Supplement: S1 Appendix — (DOCX) [file pone.0338603.s005.docx]

**TINKERCAD CIRCUIT DIAGRAM**

**
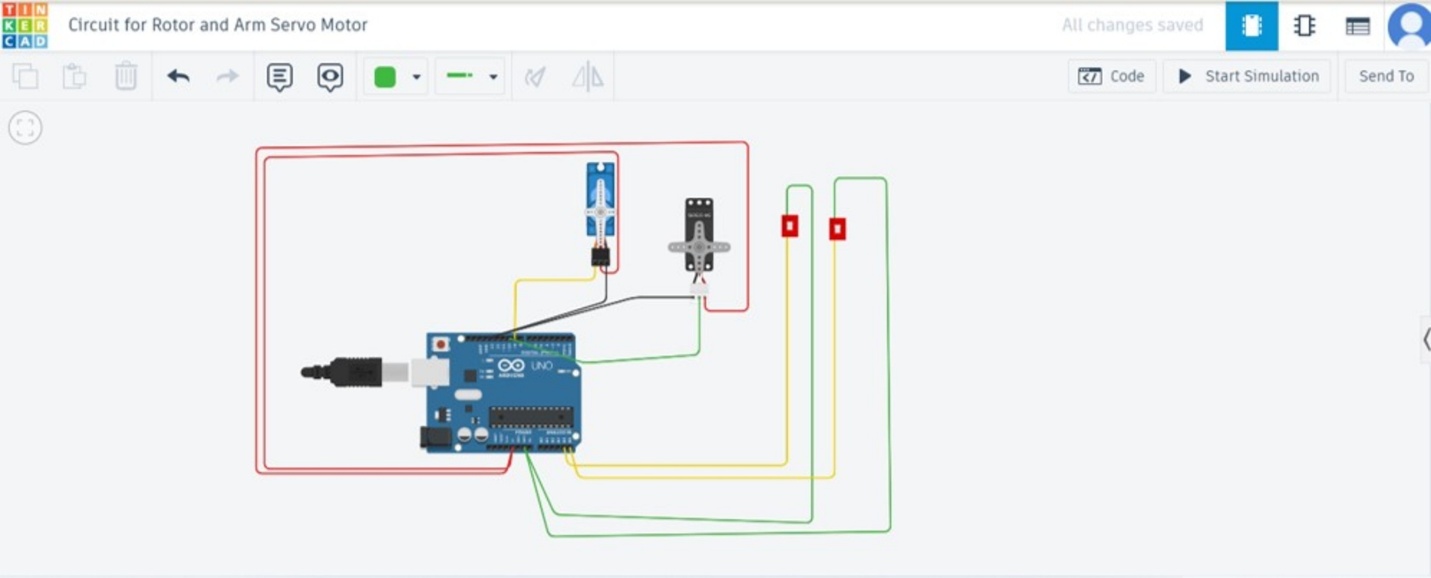
**

**Fig. Schematic of TINKERCAD circuit Diagram**

A circuit was designed and simulated in TINKERCAD to observe the movement of the arm servo (S1) and rotor servo (S2).

This approach verified that the system operated correctly based on the uploaded microcontroller code. Although the simulation in TINKERCAD employed an Arduino UNO, the prototype incorporated an Arduino Mega. The circuit was designed using Arduino UNO, two Servo Motors, and two switches. These switches act as a limit switch. This is similar to the limit switches of the weft catcher. When the A4 Switch is it represents that the weft catcher is in its forwardmost position. Then A5 is on, representing the weft catching incident by the catcher. Now the Arm Servo (black) rotates 90 degrees, similar to taking down the weft yarn. Then, depending on the weft feeder position (feeder no.) intelligent algorithm synchronizes the rotor.
