# Supplementary material for: Development of a mechatronic weft selector to enhance patterning capacity in Rapier looms
Source: PLoS One. 2025 Dec 9;20(12):e0338603. doi: 10.1371/journal.pone.0338603 (PMC12688087; doi:10.1371/journal.pone.0338603)
Supplement: S2 Appendix — (DOCX) [file pone.0338603.s006.docx]

**Weft Selection and Insertion Rate**

Miss-pick rate = $\frac{Number of miss-picks}{Total number of picks}$ × 100%

Here, 100 picks are inserted for each yarn. So, Miss-pick rate = $\frac{Number of miss-picks}{100}$ × 100%

**40 Ne Carded Cotton**

| **Types and count of yarn** | **Trial No.** | **Feeder No.** | **Selected by the Weft Selector** | **Gripped by the rapier** | **Comments** |
| --- | --- | --- | --- | --- | --- |
| 40 Ne Carded Cotton | 1 | Feeder 1 | Yes | Yes |  |
|  |  | Feeder 2 | Yes | Yes |  |
|  |  | Feeder 3 | Yes | Yes |  |
|  |  | Feeder 4 | Yes | Yes |  |
|  |  | Feeder 5 | Yes | Yes |  |
|  |  | Feeder 6 | Yes | Yes |  |
|  |  | Feeder 7 | Yes | Yes |  |
|  |  | Feeder 8 | Yes | Yes |  |
|  |  | Feeder 9 | Yes | Yes |  |
|  |  | Feeder 10 | Yes | Yes |  |
|  |  | Feeder 11 | Yes | Yes |  |
|  |  | Feeder 12 | Yes | Yes |  |
|  |  | Feeder 13 | Yes | Yes |  |
|  |  | Feeder 14 | Yes | Yes |  |
|  |  | Feeder 15 | Yes | Yes |  |
|  |  | Feeder 16 | Yes | Yes |  |
|  |  | Feeder 17 | Yes | Yes |  |
|  |  | Feeder 18 | Yes | Yes |  |
|  |  | Feeder 19 | Yes | Yes |  |
|  |  | Feeder 20 | Yes | Yes |  |
|  | | Number of wefts used = 20 | Number of successful weft selections =20 | Number of successful weft insertions =20 | Number of miss-picks =0 |
| **Result for Trial 1(40 Ne Carded Cotton)**  Weft Selection Rate for trial 1(40 Ne Carded Cotton)  = $\frac{Number of successful selections by the Weft Selector}{Number of wefts used}$ × 100%  =$\frac{20}{20}$× 100%  =100%  Pick Insertion Rate for trial 1(40 Ne Carded Cotton)  = $\frac{Number of successful weft insertion by the rapier}{Number of wefts used}$× 100%  =$\frac{20}{20}$× 100%  =100% | | | | | |

| **Types and count of yarn** | **Trial No.** | **Feeder No.** | **Selected by the Weft Selector** | **Gripped by the rapier** | **Comments** |
| --- | --- | --- | --- | --- | --- |
| 40 Ne Carded Cotton | 2 | Feeder 1 | Yes | Yes |  |
|  |  | Feeder 2 | Yes | Yes |  |
|  |  | Feeder 3 | Yes | Yes |  |
|  |  | Feeder 4 | Yes | Yes |  |
|  |  | Feeder 5 | Yes | Yes |  |
|  |  | Feeder 6 | Yes | Yes |  |
|  |  | Feeder 7 | Yes | Yes |  |
|  |  | Feeder 8 | Yes | Yes |  |
|  |  | Feeder 9 | Yes | Yes |  |
|  |  | Feeder 10 | Yes | Yes |  |
|  |  | Feeder 11 | Yes | Yes |  |
|  |  | Feeder 12 | Yes | Yes |  |
|  |  | Feeder 13 | Yes | Yes |  |
|  |  | Feeder 14 | Yes | Yes |  |
|  |  | Feeder 15 | Yes | Yes |  |
|  |  | Feeder 16 | Yes | Yes |  |
|  |  | Feeder 17 | Yes | Yes |  |
|  |  | Feeder 18 | Yes | Yes |  |
|  |  | Feeder 19 | Yes | Yes |  |
|  |  | Feeder 20 | Yes | Yes |  |
|  | | Number of wefts used = 20 | Number of successful weft selections =20 | Number of successful weft insertions =20 | Number of miss-picks =0 |
| **Result for Trial 2(40 Ne Carded Cotton)**  Weft Selection Rate for trial 2(40 Ne Carded Cotton)  = $\frac{Number of successful selections by the Weft Selector}{Number of wefts used}$ × 100%  =$\frac{20}{20}$× 100%  =100%  Pick Insertion Rate for trial 2(40 Ne Carded Cotton)  = $\frac{Number of successful weft insertion by the rapier}{Number of wefts used}$× 100%  =$\frac{20}{20}$× 100%  =100% | | | | | |

| **Types and count of yarn** | **Trial No.** | **Feeder No.** | **Selected by the Weft Selector** | **Gripped by the rapier** | **Comments** |
| --- | --- | --- | --- | --- | --- |
| 40 Ne Carded Cotton | 3 | Feeder 1 | Yes | Yes |  |
|  |  | Feeder 2 | Yes | Yes |  |
|  |  | Feeder 3 | Yes | Yes |  |
|  |  | Feeder 4 | Yes | Yes |  |
|  |  | Feeder 5 | Yes | Yes |  |
|  |  | Feeder 6 | Yes | Yes |  |
|  |  | Feeder 7 | No | No | Missed by the selector |
|  |  | Feeder 8 | Yes | Yes |  |
|  |  | Feeder 9 | Yes | Yes |  |
|  |  | Feeder 10 | Yes | Yes |  |
|  |  | Feeder 11 | Yes | Yes |  |
|  |  | Feeder 12 | Yes | Yes |  |
|  |  | Feeder 13 | Yes | Yes |  |
|  |  | Feeder 14 | Yes | Yes |  |
|  |  | Feeder 15 | Yes | Yes |  |
|  |  | Feeder 16 | Yes | Yes |  |
|  |  | Feeder 17 | Yes | Yes |  |
|  |  | Feeder 18 | Yes | Yes |  |
|  |  | Feeder 19 | Yes | Yes |  |
|  |  | Feeder 20 | Yes | Yes |  |
|  | | Number of wefts used = 20 | Number of successful weft selections =19 | Number of successful weft insertions =19 | Number of miss-picks =1 |
| **Result for Trial 3(40 Ne Carded Cotton)**  Weft Selection Rate for trial 3(40 Ne Carded Cotton)  = $\frac{Number of successful selections by the Weft Selector}{Number of wefts used}$ × 100%  =$\frac{19}{20}$× 100%  =95%  Pick Insertion Rate for trial 3(40 Ne Carded Cotton)  = $\frac{Number of successful weft insertion by the rapier}{Number of wefts used}$× 100%  =$\frac{19}{20}$× 100%  =95% | | | | | |

| **Types and count of yarn** | **Trial No.** | **Feeder No.** | **Selected by the Weft Selector** | **Gripped by the rapier** | **Comments** |
| --- | --- | --- | --- | --- | --- |
| 40 Ne Carded Cotton | 4 | Feeder 1 | Yes | Yes |  |
|  |  | Feeder 2 | Yes | Yes |  |
|  |  | Feeder 3 | Yes | Yes |  |
|  |  | Feeder 4 | Yes | Yes |  |
|  |  | Feeder 5 | Yes | Yes |  |
|  |  | Feeder 6 | Yes | Yes |  |
|  |  | Feeder 7 | Yes | Yes |  |
|  |  | Feeder 8 | Yes | Yes |  |
|  |  | Feeder 9 | Yes | Yes |  |
|  |  | Feeder 10 | Yes | Yes |  |
|  |  | Feeder 11 | Yes | Yes |  |
|  |  | Feeder 12 | Yes | Yes |  |
|  |  | Feeder 13 | Yes | Yes |  |
|  |  | Feeder 14 | Yes | Yes |  |
|  |  | Feeder 15 | Yes | Yes |  |
|  |  | Feeder 16 | Yes | Yes |  |
|  |  | Feeder 17 | Yes | Yes |  |
|  |  | Feeder 18 | Yes | Yes |  |
|  |  | Feeder 19 | Yes | Yes |  |
|  |  | Feeder 20 | Yes | Yes |  |
|  | | Number of wefts used = 20 | Number of successful weft selections =20 | Number of successful weft insertions =20 | Number of miss-picks =0 |
| **Result for Trial 4(40 Ne Carded Cotton)**  Weft Selection Rate for trial 4(40 Ne Carded Cotton)  = $\frac{Number of successful selections by the Weft Selector}{Number of wefts used}$ × 100%  =$\frac{20}{20}$× 100%  =100%  Pick Insertion Rate for trial 4(40 Ne Carded Cotton)  = $\frac{Number of successful weft insertion by the rapier}{Number of wefts used}$× 100%  =$\frac{20}{20}$× 100%  =100% | | | | | |

| **Types and count of yarn** | **Trial No.** | **Feeder No.** | **Selected by the Weft Selector** | **Gripped by the rapier** | **Comments** |
| --- | --- | --- | --- | --- | --- |
| 40 Ne Carded Cotton | 5 | Feeder 1 | Yes | Yes |  |
|  |  | Feeder 2 | Yes | Yes |  |
|  |  | Feeder 3 | Yes | Yes |  |
|  |  | Feeder 4 | Yes | Yes |  |
|  |  | Feeder 5 | Yes | Yes |  |
|  |  | Feeder 6 | Yes | Yes |  |
|  |  | Feeder 7 | Yes | Yes |  |
|  |  | Feeder 8 | Yes | Yes |  |
|  |  | Feeder 9 | Yes | Yes |  |
|  |  | Feeder 10 | Yes | Yes |  |
|  |  | Feeder 11 | Yes | Yes |  |
|  |  | Feeder 12 | Yes | Yes |  |
|  |  | Feeder 13 | Yes | Yes |  |
|  |  | Feeder 14 | Yes | Yes |  |
|  |  | Feeder 15 | Yes | Yes |  |
|  |  | Feeder 16 | Yes | Yes |  |
|  |  | Feeder 17 | Yes | Yes |  |
|  |  | Feeder 18 | Yes | Yes |  |
|  |  | Feeder 19 | Yes | Yes |  |
|  |  | Feeder 20 | Yes | Yes |  |
|  | | Number of wefts used = 20 | Number of successful weft selections =20 | Number of successful weft insertions =20 | Number of miss-picks =0 |
| **Result for Trial 5(40 Ne Carded Cotton)**  Weft Selection Rate for trial 5(40 Ne Carded Cotton)  = $\frac{Number of successful selections by the Weft Selector}{Number of wefts used}$ × 100%  =$\frac{20}{20}$× 100%  =100%  Pick Insertion Rate for trial 5(40 Ne Carded Cotton)  = $\frac{Number of successful weft insertion by the rapier}{Number of wefts used}$× 100%  =$\frac{20}{20}$× 100%  =100% | | | | | |

**Average rate of weft selection, pick insertion and Miss-Pick for 40 Ne Carded Cotton**

| **Trial No.** | **Weft Selection Rate** | **Pick Insertion Rate** | **Number of mis-picks** |
| --- | --- | --- | --- |
| 1 | 100% | 100% | 0 |
| 2 | 100% | 100% | 0 |
| 3 | 95% | 95% | 1 |
| 4 | 100% | 100% | 0 |
| 5 | 100% | 100% | 0 |
|  | Average rate of weft selection = 99% | Average rate of pick insertion =99% | Average Miss-Pick Rate= (1÷100) ×100%=1% |

**6 Ne Open end**

| **Types and count of yarn** | **Trial No.** | **Feeder No.** | **Selected by the Weft Selector** | **Gripped by the rapier** | **Comments** |
| --- | --- | --- | --- | --- | --- |
| 6 Ne Open end | 1 | Feeder 1 | Yes | Yes |  |
|  |  | Feeder 2 | Yes | Yes |  |
|  |  | Feeder 3 | Yes | Yes |  |
|  |  | Feeder 4 | Yes | Yes |  |
|  |  | Feeder 5 | Yes | Yes |  |
|  |  | Feeder 6 | Yes | Yes |  |
|  |  | Feeder 7 | Yes | Yes |  |
|  |  | Feeder 8 | Yes | Yes |  |
|  |  | Feeder 9 | Yes | Yes |  |
|  |  | Feeder 10 | Yes | Yes |  |
|  |  | Feeder 11 | Yes | Yes |  |
|  |  | Feeder 12 | Yes | Yes |  |
|  |  | Feeder 13 | Yes | Yes |  |
|  |  | Feeder 14 | Yes | Yes |  |
|  |  | Feeder 15 | Yes | Yes |  |
|  |  | Feeder 16 | Yes | Yes |  |
|  |  | Feeder 17 | Yes | Yes |  |
|  |  | Feeder 18 | Yes | Yes |  |
|  |  | Feeder 19 | Yes | Yes |  |
|  |  | Feeder 20 | Yes | Yes |  |
|  | | Number of wefts used = 20 | Number of successful weft selections =20 | Number of successful weft insertions =20 | Number of miss-picks =0 |
| **Result for Trial 1(6 Ne Open end)**  Weft Selection Rate for trial 1(6 Ne Open end)  = $\frac{Number of successful selections by the Weft Selector}{Number of wefts used}$ × 100%  =$\frac{20}{20}$× 100%  =100%  Pick Insertion Rate for trial 1(6 Ne Open end)  = $\frac{Number of successful weft insertion by the rapier}{Number of wefts used}$× 100%  =$\frac{20}{20}$× 100%  =100% | | | | | |

| **Types and count of yarn** | **Trial No.** | **Feeder No.** | **Selected by the Weft Selector** | **Gripped by the rapier** | **Comments** |
| --- | --- | --- | --- | --- | --- |
| 6 Ne Open end | 2 | Feeder 1 | Yes | Yes |  |
|  |  | Feeder 2 | Yes | Yes |  |
|  |  | Feeder 3 | Yes | Yes |  |
|  |  | Feeder 4 | Yes | Yes |  |
|  |  | Feeder 5 | Yes | Yes |  |
|  |  | Feeder 6 | Yes | Yes |  |
|  |  | Feeder 7 | Yes | Yes |  |
|  |  | Feeder 8 | Yes | Yes |  |
|  |  | Feeder 9 | Yes | Yes |  |
|  |  | Feeder 10 | Yes | Yes |  |
|  |  | Feeder 11 | Yes | Yes |  |
|  |  | Feeder 12 | Yes | Yes |  |
|  |  | Feeder 13 | Yes | Yes |  |
|  |  | Feeder 14 | Yes | Yes |  |
|  |  | Feeder 15 | Yes | Yes |  |
|  |  | Feeder 16 | Yes | Yes |  |
|  |  | Feeder 17 | Yes | Yes |  |
|  |  | Feeder 18 | Yes | Yes |  |
|  |  | Feeder 19 | Yes | Yes |  |
|  |  | Feeder 20 | Yes | Yes |  |
|  | | Number of wefts used = 20 | Number of successful weft selections =20 | Number of successful weft insertions =20 | Number of miss-picks =0 |
| **Result for Trial 2(6 Ne Open end)**  Weft Selection Rate for trial 2(6 Ne Open end)  = $\frac{Number of successful selections by the Weft Selector}{Number of wefts used}$ × 100%  =$\frac{20}{20}$× 100%  =100%  Pick Insertion Rate for trial 2(6 Ne Open end)  = $\frac{Number of successful weft insertion by the rapier}{Number of wefts used}$× 100%  =$\frac{20}{20}$× 100%  =100% | | | | | |

| **Types and count of yarn** | **Trial No.** | **Feeder No.** | **Selected by the Weft Selector** | **Gripped by the rapier** | **Comments** |
| --- | --- | --- | --- | --- | --- |
| 6 Ne Open end | 3 | Feeder 1 | Yes | Yes |  |
|  |  | Feeder 2 | Yes | Yes |  |
|  |  | Feeder 3 | Yes | Yes |  |
|  |  | Feeder 4 | Yes | Yes |  |
|  |  | Feeder 5 | Yes | Yes |  |
|  |  | Feeder 6 | Yes | Yes |  |
|  |  | Feeder 7 | Yes | Yes |  |
|  |  | Feeder 8 | Yes | Yes |  |
|  |  | Feeder 9 | Yes | Yes |  |
|  |  | Feeder 10 | Yes | Yes |  |
|  |  | Feeder 11 | Yes | Yes |  |
|  |  | Feeder 12 | Yes | Yes |  |
|  |  | Feeder 13 | Yes | Yes |  |
|  |  | Feeder 14 | Yes | Yes |  |
|  |  | Feeder 15 | Yes | Yes |  |
|  |  | Feeder 16 | Yes | Yes |  |
|  |  | Feeder 17 | Yes | Yes |  |
|  |  | Feeder 18 | Yes | Yes |  |
|  |  | Feeder 19 | Yes | Yes |  |
|  |  | Feeder 20 | Yes | Yes |  |
|  | | Number of wefts used = 20 | Number of successful weft selections =20 | Number of successful weft insertions =20 | Number of miss-picks =0 |
| **Result for Trial 3(6 Ne Open end)**  Weft Selection Rate for trial 3(6 Ne Open end)  = $\frac{Number of successful selections by the Weft Selector}{Number of wefts used}$ × 100%  =$\frac{20}{20}$× 100%  =100%  Pick Insertion Rate for trial 3(6 Ne Open end)  = $\frac{Number of successful weft insertion by the rapier}{Number of wefts used}$× 100%  =$\frac{20}{20}$× 100%  =100% | | | | | |

| **Types and count of yarn** | **Trial No.** | **Feeder No.** | **Selected by the Weft Selector** | **Gripped by the rapier** | **Comments** |
| --- | --- | --- | --- | --- | --- |
| 6 Ne Open end | 4 | Feeder 1 | Yes | Yes |  |
|  |  | Feeder 2 | Yes | Yes |  |
|  |  | Feeder 3 | Yes | Yes |  |
|  |  | Feeder 4 | Yes | Yes |  |
|  |  | Feeder 5 | Yes | Yes |  |
|  |  | Feeder 6 | Yes | Yes |  |
|  |  | Feeder 7 | Yes | Yes |  |
|  |  | Feeder 8 | Yes | Yes |  |
|  |  | Feeder 9 | Yes | Yes |  |
|  |  | Feeder 10 | Yes | Yes |  |
|  |  | Feeder 11 | Yes | Yes |  |
|  |  | Feeder 12 | Yes | Yes |  |
|  |  | Feeder 13 | Yes | Yes |  |
|  |  | Feeder 14 | Yes | Yes |  |
|  |  | Feeder 15 | Yes | Yes |  |
|  |  | Feeder 16 | Yes | Yes |  |
|  |  | Feeder 17 | Yes | Yes |  |
|  |  | Feeder 18 | Yes | Yes |  |
|  |  | Feeder 19 | Yes | Yes |  |
|  |  | Feeder 20 | Yes | Yes |  |
|  | | Number of wefts used = 20 | Number of successful weft selections =20 | Number of successful weft insertions =20 | Number of miss-picks =0 |
| **Result for Trial 4(6 Ne Open end)**  Weft Selection Rate for trial 4(6 Ne Open end)  = $\frac{Number of successful selections by the Weft Selector}{Number of wefts used}$ × 100%  =$\frac{20}{20}$× 100%  =100%  Pick Insertion Rate for trial 4(6 Ne Open end)  = $\frac{Number of successful weft insertion by the rapier}{Number of wefts used}$× 100%  =$\frac{20}{20}$× 100%  =100% | | | | | |

| **Types and count of yarn** | **Trial No.** | **Feeder No.** | **Selected by the Weft Selector** | **Gripped by the rapier** | **Comments** |
| --- | --- | --- | --- | --- | --- |
| 6 Ne Open end | 5 | Feeder 1 | Yes | Yes |  |
|  |  | Feeder 2 | Yes | Yes |  |
|  |  | Feeder 3 | Yes | Yes |  |
|  |  | Feeder 4 | Yes | Yes |  |
|  |  | Feeder 5 | Yes | Yes |  |
|  |  | Feeder 6 | Yes | Yes |  |
|  |  | Feeder 7 | Yes | Yes |  |
|  |  | Feeder 8 | Yes | Yes |  |
|  |  | Feeder 9 | Yes | Yes |  |
|  |  | Feeder 10 | Yes | Yes |  |
|  |  | Feeder 11 | Yes | Yes |  |
|  |  | Feeder 12 | Yes | Yes |  |
|  |  | Feeder 13 | Yes | Yes |  |
|  |  | Feeder 14 | Yes | Yes |  |
|  |  | Feeder 15 | Yes | Yes |  |
|  |  | Feeder 16 | Yes | Yes |  |
|  |  | Feeder 17 | Yes | Yes |  |
|  |  | Feeder 18 | Yes | Yes |  |
|  |  | Feeder 19 | Yes | Yes |  |
|  |  | Feeder 20 | Yes | Yes |  |
|  | | Number of wefts used = 20 | Number of successful weft selections =20 | Number of successful weft insertions =20 | Number of miss-picks =0 |
| **Result for Trial 5(6 Ne Open end)**  Weft Selection Rate for trial 5(6 Ne Open end)  = $\frac{Number of successful selections by the Weft Selector}{Number of wefts used}$ × 100%  =$\frac{20}{20}$× 100%  =100%  Pick Insertion Rate for trial 5(20 Ne Open end)  = $\frac{Number of successful weft insertion by the rapier}{Number of wefts used}$× 100%  =$\frac{20}{20}$× 100%  =100% | | | | | |

**Average rate of weft selection, pick insertion, and Miss-Pick for 6 Ne Carded Cotton**

| **Trial No.** | **Weft Selection Rate** | **Pick Insertion Rate** | **Number of mis-picks** |
| --- | --- | --- | --- |
| 1 | 100% | 100% | 0 |
| 2 | 100% | 100% | 0 |
| 3 | 100% | 100% | 0 |
| 4 | 100% | 100% | 0 |
| 5 | 100% | 100% | 0 |
|  | Average rate of weft selection = 100% | Average rate of pick insertion =100% | Average Miss-Pick Rate= (0÷100) ×100%=0% |

**20 Ne Open end**

| **Types and count of yarn** | **Trial No.** | **Feeder No.** | **Selected by the Weft Selector** | **Gripped by the rapier** | **Comments** |
| --- | --- | --- | --- | --- | --- |
| 20 Ne Open end | 1 | Feeder 1 | Yes | Yes |  |
|  |  | Feeder 2 | Yes | Yes |  |
|  |  | Feeder 3 | Yes | Yes |  |
|  |  | Feeder 4 | Yes | Yes |  |
|  |  | Feeder 5 | Yes | Yes |  |
|  |  | Feeder 6 | Yes | Yes |  |
|  |  | Feeder 7 | Yes | Yes |  |
|  |  | Feeder 8 | Yes | Yes |  |
|  |  | Feeder 9 | Yes | Yes |  |
|  |  | Feeder 10 | Yes | Yes |  |
|  |  | Feeder 11 | Yes | Yes |  |
|  |  | Feeder 12 | Yes | Yes |  |
|  |  | Feeder 13 | Yes | Yes |  |
|  |  | Feeder 14 | Yes | Yes |  |
|  |  | Feeder 15 | Yes | Yes |  |
|  |  | Feeder 16 | Yes | Yes |  |
|  |  | Feeder 17 | Yes | Yes |  |
|  |  | Feeder 18 | Yes | Yes |  |
|  |  | Feeder 19 | Yes | Yes |  |
|  |  | Feeder 20 | Yes | Yes |  |
|  | | Number of wefts used = 20 | Number of successful weft selections =20 | Number of successful weft insertions =20 | Number of miss-picks =0 |
| **Result for Trial 1(20 Ne Open end)**  Weft Selection Rate for trial 1(20 Ne Open end)  = $\frac{Number of successful selections by the Weft Selector}{Number of wefts used}$ × 100%  =$\frac{20}{20}$× 100%  =100%  Pick Insertion Rate for trial 1(20 Ne Open end)  = $\frac{Number of successful weft insertion by the rapier}{Number of wefts used}$× 100%  =$\frac{20}{20}$× 100%  =100% | | | | | |

| **Types and count of yarn** | **Trial No.** | **Feeder No.** | **Selected by the Weft Selector** | **Gripped by the rapier** | **Comments** |
| --- | --- | --- | --- | --- | --- |
| 20 Ne Open end | 2 | Feeder 1 | Yes | Yes |  |
|  |  | Feeder 2 | Yes | Yes |  |
|  |  | Feeder 3 | Yes | Yes |  |
|  |  | Feeder 4 | Yes | Yes |  |
|  |  | Feeder 5 | Yes | Yes |  |
|  |  | Feeder 6 | Yes | Yes |  |
|  |  | Feeder 7 | Yes | Yes |  |
|  |  | Feeder 8 | Yes | Yes |  |
|  |  | Feeder 9 | Yes | Yes |  |
|  |  | Feeder 10 | Yes | Yes |  |
|  |  | Feeder 11 | Yes | Yes |  |
|  |  | Feeder 12 | Yes | Yes |  |
|  |  | Feeder 13 | Yes | Yes |  |
|  |  | Feeder 14 | Yes | Yes |  |
|  |  | Feeder 15 | Yes | Yes |  |
|  |  | Feeder 16 | Yes | Yes |  |
|  |  | Feeder 17 | Yes | Yes |  |
|  |  | Feeder 18 | Yes | Yes |  |
|  |  | Feeder 19 | Yes | Yes |  |
|  |  | Feeder 20 | Yes | Yes |  |
|  | | Number of wefts used = 20 | Number of successful weft selections =20 | Number of successful weft insertions =20 | Number of miss-picks =0 |
| **Result for Trial 2(20 Ne Open end)**  Weft Selection Rate for trial 2(20 Ne Open end)  = $\frac{Number of successful selections by the Weft Selector}{Number of wefts used}$ × 100%  =$\frac{20}{20}$× 100%  =100%  Pick Insertion Rate for trial 2(20 Ne Open end)  = $\frac{Number of successful weft insertion by the rapier}{Number of wefts used}$× 100%  =$\frac{20}{20}$× 100%  =100% | | | | | |

| **Types and count of yarn** | **Trial No.** | **Feeder No.** | **Selected by the Weft Selector** | **Gripped by the rapier** | **Comments** |
| --- | --- | --- | --- | --- | --- |
| 20 Ne Open end | 3 | Feeder 1 | Yes | Yes |  |
|  |  | Feeder 2 | Yes | Yes |  |
|  |  | Feeder 3 | Yes | Yes |  |
|  |  | Feeder 4 | Yes | Yes |  |
|  |  | Feeder 5 | Yes | Yes |  |
|  |  | Feeder 6 | Yes | Yes |  |
|  |  | Feeder 7 | Yes | Yes |  |
|  |  | Feeder 8 | Yes | Yes |  |
|  |  | Feeder 9 | Yes | Yes |  |
|  |  | Feeder 10 | Yes | Yes |  |
|  |  | Feeder 11 | Yes | Yes |  |
|  |  | Feeder 12 | Yes | Yes |  |
|  |  | Feeder 13 | Yes | Yes |  |
|  |  | Feeder 14 | Yes | Yes |  |
|  |  | Feeder 15 | Yes | Yes |  |
|  |  | Feeder 16 | Yes | Yes |  |
|  |  | Feeder 17 | Yes | Yes |  |
|  |  | Feeder 18 | Yes | Yes |  |
|  |  | Feeder 19 | Yes | Yes |  |
|  |  | Feeder 20 | Yes | Yes |  |
|  | | Number of wefts used = 20 | Number of successful weft selections =20 | Number of successful weft insertions =20 | Number of miss-picks =0 |
| **Result for Trial 3(20 Ne Open end)**  Weft Selection Rate for trial 3(20 Ne Open end)  = $\frac{Number of successful selections by the Weft Selector}{Number of wefts used}$ × 100%  =$\frac{20}{20}$× 100%  =100%  Pick Insertion Rate for trial 3(20 Ne Open end)  = $\frac{Number of successful weft insertion by the rapier}{Number of wefts used}$× 100%  =$\frac{20}{20}$× 100%  =100% | | | | | |

| **Types and count of yarn** | **Trial No.** | **Feeder No.** | **Selected by the Weft Selector** | **Gripped by the rapier** | **Comments** |
| --- | --- | --- | --- | --- | --- |
| 20 Ne Open end | 4 | Feeder 1 | Yes | Yes |  |
|  |  | Feeder 2 | Yes | Yes |  |
|  |  | Feeder 3 | Yes | Yes |  |
|  |  | Feeder 4 | Yes | Yes |  |
|  |  | Feeder 5 | Yes | Yes |  |
|  |  | Feeder 6 | Yes | Yes |  |
|  |  | Feeder 7 | Yes | Yes |  |
|  |  | Feeder 8 | Yes | Yes |  |
|  |  | Feeder 9 | Yes | Yes |  |
|  |  | Feeder 10 | Yes | Yes |  |
|  |  | Feeder 11 | Yes | Yes |  |
|  |  | Feeder 12 | Yes | Yes |  |
|  |  | Feeder 13 | Yes | Yes |  |
|  |  | Feeder 14 | Yes | Yes |  |
|  |  | Feeder 15 | Yes | Yes |  |
|  |  | Feeder 16 | Yes | Yes |  |
|  |  | Feeder 17 | Yes | Yes |  |
|  |  | Feeder 18 | Yes | Yes |  |
|  |  | Feeder 19 | Yes | Yes |  |
|  |  | Feeder 20 | Yes | Yes |  |
|  | | Number of wefts used = 20 | Number of successful weft selections =20 | Number of successful weft insertions =20 | Number of miss-picks =0 |
| **Result for Trial 4(20 Ne Open end)**  Weft Selection Rate for trial 4(20 Ne Open end)  = $\frac{Number of successful selections by the Weft Selector}{Number of wefts used}$ × 100%  =$\frac{20}{20}$× 100%  =100%  Pick Insertion Rate for trial 4(20 Ne Open end)  = $\frac{Number of successful weft insertion by the rapier}{Number of wefts used}$× 100%  =$\frac{20}{20}$× 100%  =100% | | | | | |

| **Types and count of yarn** | **Trial No.** | **Feeder No.** | **Selected by the Weft Selector** | **Gripped by the rapier** | **Comments** |
| --- | --- | --- | --- | --- | --- |
| 20 Ne Open end | 5 | Feeder 1 | Yes | Yes |  |
|  |  | Feeder 2 | Yes | Yes |  |
|  |  | Feeder 3 | Yes | Yes |  |
|  |  | Feeder 4 | Yes | Yes |  |
|  |  | Feeder 5 | Yes | Yes |  |
|  |  | Feeder 6 | Yes | Yes |  |
|  |  | Feeder 7 | Yes | Yes |  |
|  |  | Feeder 8 | Yes | Yes |  |
|  |  | Feeder 9 | Yes | Yes |  |
|  |  | Feeder 10 | Yes | Yes |  |
|  |  | Feeder 11 | Yes | Yes |  |
|  |  | Feeder 12 | Yes | Yes |  |
|  |  | Feeder 13 | Yes | Yes |  |
|  |  | Feeder 14 | Yes | Yes |  |
|  |  | Feeder 15 | Yes | Yes |  |
|  |  | Feeder 16 | Yes | Yes |  |
|  |  | Feeder 17 | Yes | Yes |  |
|  |  | Feeder 18 | Yes | Yes |  |
|  |  | Feeder 19 | Yes | Yes |  |
|  |  | Feeder 20 | Yes | Yes |  |
|  | | Number of wefts used = 20 | Number of successful weft selections =20 | Number of successful weft insertions =20 | Number of miss-picks =0 |
| **Result for Trial 5(20 Ne Open end)**  Weft Selection Rate for trial 5(20 Ne Open end)  = $\frac{Number of successful selections by the Weft Selector}{Number of wefts used}$ × 100%  =$\frac{20}{20}$× 100%  =100%  Pick Insertion Rate for trial 5(20 Ne Open end)  = $\frac{Number of successful weft insertion by the rapier}{Number of wefts used}$× 100%  =$\frac{20}{20}$× 100%  =100% | | | | | |

**Average rate of weft selection, pick insertion, and Miss-Pick for 20 Ne Open end**

| **Trial No.** | **Weft Selection Rate** | **Pick Insertion Rate** | **Number of mis-picks** |
| --- | --- | --- | --- |
| 1 | 100% | 100% | 0 |
| 2 | 100% | 100% | 0 |
| 3 | 100% | 100% | 0 |
| 4 | 100% | 100% | 0 |
| 5 | 100% | 100% | 0 |
|  | Average rate of weft selection = 100% | Average rate of pick insertion =100% | Average Miss-Pick Rate= (0÷100) ×100%=0% |

**20 Ne Viscose**

| **Types and count of yarn** | **Trial No.** | **Feeder No.** | **Selected by the Weft Selector** | **Gripped by the rapier** | **Comments** |
| --- | --- | --- | --- | --- | --- |
| 20 Ne Viscose | 1 | Feeder 1 | Yes | Yes |  |
|  |  | Feeder 2 | Yes | Yes |  |
|  |  | Feeder 3 | Yes | Yes |  |
|  |  | Feeder 4 | Yes | Yes |  |
|  |  | Feeder 5 | Yes | Yes |  |
|  |  | Feeder 6 | Yes | Yes |  |
|  |  | Feeder 7 | Yes | Yes |  |
|  |  | Feeder 8 | Yes | Yes |  |
|  |  | Feeder 9 | Yes | Yes |  |
|  |  | Feeder 10 | Yes | Yes |  |
|  |  | Feeder 11 | Yes | Yes |  |
|  |  | Feeder 12 | Yes | Yes |  |
|  |  | Feeder 13 | Yes | Yes |  |
|  |  | Feeder 14 | Yes | Yes |  |
|  |  | Feeder 15 | Yes | Yes |  |
|  |  | Feeder 16 | Yes | Yes |  |
|  |  | Feeder 17 | Yes | Yes |  |
|  |  | Feeder 18 | Yes | Yes |  |
|  |  | Feeder 19 | Yes | Yes |  |
|  |  | Feeder 20 | Yes | Yes |  |
|  | | Number of wefts used = 20 | Number of successful weft selections =20 | Number of successful weft insertions =20 | Number of miss-picks =0 |
| **Result for Trial 1(20 Ne Viscose)**  Weft Selection Rate for trial 1(20 Ne Viscose)  = $\frac{Number of successful selections by the Weft Selector}{Number of wefts used}$ × 100%  =$\frac{20}{20}$× 100%  =100%  Pick Insertion Rate for trial 1(20 Ne Viscose)  = $\frac{Number of successful weft insertion by the rapier}{Number of wefts used}$× 100%  =$\frac{20}{20}$× 100%  =100% | | | | | |

| **Types and count of yarn** | **Trial No.** | **Feeder No.** | **Selected by the Weft Selector** | **Gripped by the rapier** | **Comments** |
| --- | --- | --- | --- | --- | --- |
| 20 Ne Viscose | 2 | Feeder 1 | Yes | Yes |  |
|  |  | Feeder 2 | Yes | Yes |  |
|  |  | Feeder 3 | Yes | Yes |  |
|  |  | Feeder 4 | Yes | Yes |  |
|  |  | Feeder 5 | Yes | Yes |  |
|  |  | Feeder 6 | Yes | Yes |  |
|  |  | Feeder 7 | Yes | Yes |  |
|  |  | Feeder 8 | Yes | Yes |  |
|  |  | Feeder 9 | Yes | Yes |  |
|  |  | Feeder 10 | Yes | Yes |  |
|  |  | Feeder 11 | Yes | Yes |  |
|  |  | Feeder 12 | Yes | Yes |  |
|  |  | Feeder 13 | Yes | Yes |  |
|  |  | Feeder 14 | Yes | Yes |  |
|  |  | Feeder 15 | Yes | Yes |  |
|  |  | Feeder 16 | Yes | Yes |  |
|  |  | Feeder 17 | Yes | Yes |  |
|  |  | Feeder 18 | Yes | Yes |  |
|  |  | Feeder 19 | Yes | Yes |  |
|  |  | Feeder 20 | Yes | Yes |  |
|  | | Number of wefts used = 20 | Number of successful weft selections =20 | Number of successful weft insertions =20 | Number of miss-picks =0 |
| **Result for Trial 2(20 Ne Viscose)**  Weft Selection Rate for trial 2(20 Ne Viscose)  = $\frac{Number of successful selections by the Weft Selector}{Number of wefts used}$ × 100%  =$\frac{20}{20}$× 100%  =100%  Pick Insertion Rate for trial 2(20 Ne Viscose)  = $\frac{Number of successful weft insertion by the rapier}{Number of wefts used}$× 100%  =$\frac{20}{20}$× 100%  =100% | | | | | |

| **Types and count of yarn** | **Trial No.** | **Feeder No.** | **Selected by the Weft Selector** | **Gripped by the rapier** | **Comments** |
| --- | --- | --- | --- | --- | --- |
| 20 Ne Viscose | 3 | Feeder 1 | Yes | Yes |  |
|  |  | Feeder 2 | Yes | Yes |  |
|  |  | Feeder 3 | Yes | Yes |  |
|  |  | Feeder 4 | Yes | Yes |  |
|  |  | Feeder 5 | Yes | Yes |  |
|  |  | Feeder 6 | Yes | Yes |  |
|  |  | Feeder 7 | Yes | Yes |  |
|  |  | Feeder 8 | Yes | Yes |  |
|  |  | Feeder 9 | Yes | Yes |  |
|  |  | Feeder 10 | Yes | Yes |  |
|  |  | Feeder 11 | Yes | Yes |  |
|  |  | Feeder 12 | Yes | Yes |  |
|  |  | Feeder 13 | Yes | Yes |  |
|  |  | Feeder 14 | Yes | Yes |  |
|  |  | Feeder 15 | Yes | Yes |  |
|  |  | Feeder 16 | Yes | Yes |  |
|  |  | Feeder 17 | Yes | Yes |  |
|  |  | Feeder 18 | Yes | Yes |  |
|  |  | Feeder 19 | Yes | Yes |  |
|  |  | Feeder 20 | Yes | Yes |  |
|  | | Number of wefts used = 20 | Number of successful weft selections =20 | Number of successful weft insertions =20 | Number of miss-picks =0 |
| **Result for Trial 3(20 Ne Viscose)**  Weft Selection Rate for trial 3(20 Ne Viscose)  = $\frac{Number of successful selections by the Weft Selector}{Number of wefts used}$ × 100%  =$\frac{20}{20}$× 100%  =100%  Pick Insertion Rate for trial 3(20 Ne Viscose)  = $\frac{Number of successful weft insertion by the rapier}{Number of wefts used}$× 100%  =$\frac{20}{20}$× 100%  =100% | | | | | |

| **Types and count of yarn** | **Trial No.** | **Feeder No.** | **Selected by the Weft Selector** | **Gripped by the rapier** | **Comments** |
| --- | --- | --- | --- | --- | --- |
| 20 Ne Viscose | 4 | Feeder 1 | Yes | Yes |  |
|  |  | Feeder 2 | Yes | Yes |  |
|  |  | Feeder 3 | Yes | Yes |  |
|  |  | Feeder 4 | Yes | Yes |  |
|  |  | Feeder 5 | Yes | Yes |  |
|  |  | Feeder 6 | Yes | Yes |  |
|  |  | Feeder 7 | Yes | Yes |  |
|  |  | Feeder 8 | Yes | Yes |  |
|  |  | Feeder 9 | Yes | Yes |  |
|  |  | Feeder 10 | Yes | Yes |  |
|  |  | Feeder 11 | Yes | Yes |  |
|  |  | Feeder 12 | Yes | Yes |  |
|  |  | Feeder 13 | Yes | Yes |  |
|  |  | Feeder 14 | Yes | Yes |  |
|  |  | Feeder 15 | Yes | Yes |  |
|  |  | Feeder 16 | Yes | Yes |  |
|  |  | Feeder 17 | Yes | Yes |  |
|  |  | Feeder 18 | Yes | Yes |  |
|  |  | Feeder 19 | Yes | Yes |  |
|  |  | Feeder 20 | Yes | Yes |  |
|  | | Number of wefts used = 20 | Number of successful weft selections =20 | Number of successful weft insertions =20 | Number of miss-picks =0 |
| **Result for Trial 4(20 Ne Viscose)**  Weft Selection Rate for trial 4(20 Ne Viscose)  = $\frac{Number of successful selections by the Weft Selector}{Number of wefts used}$ × 100%  =$\frac{20}{20}$× 100%  =100%  Pick Insertion Rate for trial 4(20 Ne Viscose)  = $\frac{Number of successful weft insertion by the rapier}{Number of wefts used}$× 100%  =$\frac{20}{20}$× 100%  =100% | | | | | |

| **Types and count of yarn** | **Trial No.** | **Feeder No.** | **Selected by the Weft Selector** | **Gripped by the rapier** | **Comments** |
| --- | --- | --- | --- | --- | --- |
| 20 Ne Viscose | 5 | Feeder 1 | Yes | Yes |  |
|  |  | Feeder 2 | Yes | Yes |  |
|  |  | Feeder 3 | Yes | Yes |  |
|  |  | Feeder 4 | Yes | Yes |  |
|  |  | Feeder 5 | Yes | Yes |  |
|  |  | Feeder 6 | Yes | Yes |  |
|  |  | Feeder 7 | Yes | Yes |  |
|  |  | Feeder 8 | Yes | Yes |  |
|  |  | Feeder 9 | Yes | Yes |  |
|  |  | Feeder 10 | Yes | Yes |  |
|  |  | Feeder 11 | Yes | Yes |  |
|  |  | Feeder 12 | Yes | Yes |  |
|  |  | Feeder 13 | Yes | Yes |  |
|  |  | Feeder 14 | Yes | Yes |  |
|  |  | Feeder 15 | Yes | Yes |  |
|  |  | Feeder 16 | Yes | Yes |  |
|  |  | Feeder 17 | Yes | Yes |  |
|  |  | Feeder 18 | Yes | Yes |  |
|  |  | Feeder 19 | Yes | Yes |  |
|  |  | Feeder 20 | Yes | Yes |  |
|  | | Number of wefts used = 20 | Number of successful weft selections =20 | Number of successful weft insertions =20 | Number of miss-picks =0 |
| **Result for Trial 5(20 Ne Viscose)**  Weft Selection Rate for trial 5(20 Ne Viscose)  = $\frac{Number of successful selections by the Weft Selector}{Number of wefts used}$ × 100%  =$\frac{20}{20}$× 100%  =100%  Pick Insertion Rate for trial 5(20 Ne Viscose)  = $\frac{Number of successful weft insertion by the rapier}{Number of wefts used}$× 100%  =$\frac{20}{20}$× 100%  =100% | | | | | |

**Average rate of weft selection, pick insertion, and Miss-Pick for 20 Ne Viscose**

| **Trial No.** | **Weft Selection Rate** | **Pick Insertion Rate** | **Number of mis-picks** |
| --- | --- | --- | --- |
| 1 | 100% | 100% | 0 |
| 2 | 100% | 100% | 0 |
| 3 | 100% | 100% | 0 |
| 4 | 100% | 100% | 0 |
| 5 | 100% | 100% | 0 |
|  | Average rate of weft selection = 100% | Average rate of pick insertion =100% | Average Miss-Pick Rate= (0÷100) ×100%=0% |

**30 Ne Viscose**

| **Types and count of yarn** | **Trial No.** | **Feeder No.** | **Selected by the Weft Selector** | **Gripped by the rapier** | **Comments** |
| --- | --- | --- | --- | --- | --- |
| 30 Ne Viscose | 1 | Feeder 1 | Yes | Yes |  |
|  |  | Feeder 2 | Yes | Yes |  |
|  |  | Feeder 3 | Yes | Yes |  |
|  |  | Feeder 4 | Yes | Yes |  |
|  |  | Feeder 5 | Yes | Yes |  |
|  |  | Feeder 6 | Yes | Yes |  |
|  |  | Feeder 7 | Yes | Yes |  |
|  |  | Feeder 8 | Yes | Yes |  |
|  |  | Feeder 9 | Yes | Yes |  |
|  |  | Feeder 10 | Yes | Yes |  |
|  |  | Feeder 11 | Yes | Yes |  |
|  |  | Feeder 12 | Yes | Yes |  |
|  |  | Feeder 13 | Yes | Yes |  |
|  |  | Feeder 14 | Yes | Yes |  |
|  |  | Feeder 15 | Yes | Yes |  |
|  |  | Feeder 16 | Yes | Yes |  |
|  |  | Feeder 17 | Yes | Yes |  |
|  |  | Feeder 18 | Yes | Yes |  |
|  |  | Feeder 19 | Yes | Yes |  |
|  |  | Feeder 20 | Yes | Yes |  |
|  | | Number of wefts used = 20 | Number of successful weft selections =20 | Number of successful weft insertions =20 | Number of miss-picks =0 |
| **Result for Trial 1(30 Ne Viscose)**  Weft Selection Rate for trial 1(30 Ne Viscose)  = $\frac{Number of successful selections by the Weft Selector}{Number of wefts used}$ × 100%  =$\frac{20}{20}$× 100%  =100%  Pick Insertion Rate for trial 1(30 Ne Viscose)  = $\frac{Number of successful weft insertion by the rapier}{Number of wefts used}$× 100%  =$\frac{20}{20}$× 100%  =100% | | | | | |

| **Types and count of yarn** | **Trial No.** | **Feeder No.** | **Selected by the Weft Selector** | **Gripped by the rapier** | **Comments** |
| --- | --- | --- | --- | --- | --- |
| 30 Ne Viscose | 2 | Feeder 1 | Yes | Yes |  |
|  |  | Feeder 2 | Yes | Yes |  |
|  |  | Feeder 3 | Yes | Yes |  |
|  |  | Feeder 4 | Yes | Yes |  |
|  |  | Feeder 5 | Yes | Yes |  |
|  |  | Feeder 6 | Yes | Yes |  |
|  |  | Feeder 7 | Yes | Yes |  |
|  |  | Feeder 8 | Yes | Yes |  |
|  |  | Feeder 9 | Yes | Yes |  |
|  |  | Feeder 10 | Yes | Yes |  |
|  |  | Feeder 11 | Yes | Yes |  |
|  |  | Feeder 12 | Yes | Yes |  |
|  |  | Feeder 13 | Yes | Yes |  |
|  |  | Feeder 14 | Yes | Yes |  |
|  |  | Feeder 15 | Yes | Yes |  |
|  |  | Feeder 16 | Yes | Yes |  |
|  |  | Feeder 17 | Yes | Yes |  |
|  |  | Feeder 18 | Yes | Yes |  |
|  |  | Feeder 19 | Yes | Yes |  |
|  |  | Feeder 20 | Yes | Yes |  |
|  | | Number of wefts used = 20 | Number of successful weft selections =20 | Number of successful weft insertions =20 | Number of miss-picks =0 |
| **Result for Trial 2(30 Ne Viscose)**  Weft Selection Rate for trial 2(30 Ne Viscose)  = $\frac{Number of successful selections by the Weft Selector}{Number of wefts used}$ × 100%  =$\frac{20}{20}$× 100%  =100%  Pick Insertion Rate for trial 2(30 Ne Viscose)  = $\frac{Number of successful weft insertion by the rapier}{Number of wefts used}$× 100%  =$\frac{20}{20}$× 100%  =100% | | | | | |

| **Types and count of yarn** | **Trial No.** | **Feeder No.** | **Selected by the Weft Selector** | **Gripped by the rapier** | **Comments** |
| --- | --- | --- | --- | --- | --- |
| 30 Ne Viscose | 3 | Feeder 1 | Yes | Yes |  |
|  |  | Feeder 2 | Yes | Yes |  |
|  |  | Feeder 3 | Yes | Yes |  |
|  |  | Feeder 4 | Yes | Yes |  |
|  |  | Feeder 5 | Yes | Yes |  |
|  |  | Feeder 6 | Yes | Yes |  |
|  |  | Feeder 7 | Yes | Yes |  |
|  |  | Feeder 8 | Yes | Yes |  |
|  |  | Feeder 9 | Yes | Yes |  |
|  |  | Feeder 10 | Yes | Yes |  |
|  |  | Feeder 11 | Yes | Yes |  |
|  |  | Feeder 12 | Yes | Yes |  |
|  |  | Feeder 13 | Yes | Yes |  |
|  |  | Feeder 14 | Yes | Yes |  |
|  |  | Feeder 15 | Yes | Yes |  |
|  |  | Feeder 16 | Yes | Yes |  |
|  |  | Feeder 17 | No | No | Missed by the selector |
|  |  | Feeder 18 | Yes | Yes |  |
|  |  | Feeder 19 | Yes | Yes |  |
|  |  | Feeder 20 | Yes | Yes |  |
|  | | Number of wefts used = 20 | Number of successful weft selections =19 | Number of successful weft insertions =19 | Number of miss-picks =1 |
| **Result for Trial 3(30 Ne Viscose)**  Weft Selection Rate for trial 3(30 Ne Viscose)  = $\frac{Number of successful selections by the Weft Selector}{Number of wefts used}$ × 100%  =$\frac{19}{20}$× 100%  =95%  Pick Insertion Rate for trial 3(30 Ne Viscose)  = $\frac{Number of successful weft insertion by the rapier}{Number of wefts used}$× 100%  =$\frac{19}{20}$× 100%  =95% | | | | | |

| **Types and count of yarn** | **Trial No.** | **Feeder No.** | **Selected by the Weft Selector** | **Gripped by the rapier** | **Comments** |
| --- | --- | --- | --- | --- | --- |
| 30 Ne Viscose | 4 | Feeder 1 | Yes | Yes |  |
|  |  | Feeder 2 | Yes | Yes |  |
|  |  | Feeder 3 | Yes | Yes |  |
|  |  | Feeder 4 | Yes | Yes |  |
|  |  | Feeder 5 | Yes | Yes |  |
|  |  | Feeder 6 | Yes | Yes |  |
|  |  | Feeder 7 | Yes | Yes |  |
|  |  | Feeder 8 | Yes | Yes |  |
|  |  | Feeder 9 | Yes | Yes |  |
|  |  | Feeder 10 | Yes | Yes |  |
|  |  | Feeder 11 | Yes | Yes |  |
|  |  | Feeder 12 | Yes | Yes |  |
|  |  | Feeder 13 | Yes | Yes |  |
|  |  | Feeder 14 | Yes | Yes |  |
|  |  | Feeder 15 | Yes | Yes |  |
|  |  | Feeder 16 | Yes | Yes |  |
|  |  | Feeder 17 | Yes | Yes |  |
|  |  | Feeder 18 | Yes | Yes |  |
|  |  | Feeder 19 | Yes | Yes |  |
|  |  | Feeder 20 | Yes | Yes |  |
|  | | Number of wefts used = 20 | Number of successful weft selections =20 | Number of successful weft insertions =20 | Number of miss-picks =0 |
| **Result for Trial 4(30 Ne Viscose)**  Weft Selection Rate for trial 4(30 Ne Viscose)  = $\frac{Number of successful selections by the Weft Selector}{Number of wefts used}$ × 100%  =$\frac{20}{20}$× 100%  =100%  Pick Insertion Rate for trial 4(30 Ne Viscose)  = $\frac{Number of successful weft insertion by the rapier}{Number of wefts used}$× 100%  =$\frac{20}{20}$× 100%  =100% | | | | | |

| **Types and count of yarn** | **Trial No.** | **Feeder No.** | **Selected by the Weft Selector** | **Gripped by the rapier** | **Comments** |
| --- | --- | --- | --- | --- | --- |
| 30 Ne Viscose | 5 | Feeder 1 | Yes | Yes |  |
|  |  | Feeder 2 | Yes | Yes |  |
|  |  | Feeder 3 | Yes | Yes |  |
|  |  | Feeder 4 | Yes | Yes |  |
|  |  | Feeder 5 | Yes | Yes |  |
|  |  | Feeder 6 | Yes | Yes |  |
|  |  | Feeder 7 | Yes | Yes |  |
|  |  | Feeder 8 | Yes | Yes |  |
|  |  | Feeder 9 | Yes | Yes |  |
|  |  | Feeder 10 | Yes | Yes |  |
|  |  | Feeder 11 | Yes | Yes |  |
|  |  | Feeder 12 | Yes | Yes |  |
|  |  | Feeder 13 | Yes | Yes |  |
|  |  | Feeder 14 | Yes | Yes |  |
|  |  | Feeder 15 | Yes | Yes |  |
|  |  | Feeder 16 | Yes | Yes |  |
|  |  | Feeder 17 | Yes | Yes |  |
|  |  | Feeder 18 | Yes | Yes |  |
|  |  | Feeder 19 | Yes | Yes |  |
|  |  | Feeder 20 | Yes | Yes |  |
|  | | Number of wefts used = 20 | Number of successful weft selections =20 | Number of successful weft insertions =20 | Number of miss-picks =0 |
| **Result for Trial 5(30 Ne Viscose)**  Weft Selection Rate for trial 5(30 Ne Viscose)  = $\frac{Number of successful selections by the Weft Selector}{Number of wefts used}$ × 100%  =$\frac{20}{20}$× 100%  =100%  Pick Insertion Rate for trial 5(30 Ne Viscose)  = $\frac{Number of successful weft insertion by the rapier}{Number of wefts used}$× 100%  =$\frac{20}{20}$× 100%  =100% | | | | | |

**Average rate of weft selection, pick insertion and Miss-Pick for 30 Ne Viscose**

| **Trial No.** | **Weft Selection Rate** | **Pick Insertion Rate** | **Number of mis-picks** |
| --- | --- | --- | --- |
| 1 | 100% | 100% | 0 |
| 2 | 100% | 100% | 0 |
| 3 | 95% | 95% | 1 |
| 4 | 100% | 100% | 0 |
| 5 | 100% | 100% | 0 |
|  | Average rate of weft selection = 99% | Average rate of pick insertion =99% | Average Miss-Pick Rate= (1÷100) ×100%=1% |

**40 Ne Viscose**

| **Types and count of yarn** | **Trial No.** | **Feeder No.** | **Selected by the Weft Selector** | **Gripped by the rapier** | **Comments** |
| --- | --- | --- | --- | --- | --- |
| 40 Ne Viscose | 1 | Feeder 1 | Yes | Yes |  |
|  |  | Feeder 2 | Yes | Yes |  |
|  |  | Feeder 3 | Yes | Yes |  |
|  |  | Feeder 4 | Yes | Yes |  |
|  |  | Feeder 5 | Yes | Yes |  |
|  |  | Feeder 6 | Yes | Yes |  |
|  |  | Feeder 7 | Yes | Yes |  |
|  |  | Feeder 8 | Yes | Yes |  |
|  |  | Feeder 9 | Yes | Yes |  |
|  |  | Feeder 10 | Yes | Yes |  |
|  |  | Feeder 11 | Yes | Yes |  |
|  |  | Feeder 12 | Yes | Yes |  |
|  |  | Feeder 13 | Yes | Yes |  |
|  |  | Feeder 14 | Yes | Yes |  |
|  |  | Feeder 15 | Yes | Yes |  |
|  |  | Feeder 16 | Yes | Yes |  |
|  |  | Feeder 17 | Yes | Yes |  |
|  |  | Feeder 18 | No | No | Missed by the selector |
|  |  | Feeder 19 | Yes | Yes |  |
|  |  | Feeder 20 | Yes | Yes |  |
|  | | Number of wefts used = 20 | Number of successful weft selections =19 | Number of successful weft insertions =19 | Number of miss-picks =1 |
| **Result for Trial 1(40 Ne Viscose)**  Weft Selection Rate for trial 1(40 Ne Viscose)  = $\frac{Number of successful selections by the Weft Selector}{Number of wefts used}$ × 100%  =$\frac{19}{20}$× 100%  =95%  Pick Insertion Rate for trial 1(40 Ne Viscose)  = $\frac{Number of successful weft insertion by the rapier}{Number of wefts used}$× 100%  =$\frac{19}{20}$× 100%  =95% | | | | | |

| **Types and count of yarn** | **Trial No.** | **Feeder No.** | **Selected by the Weft Selector** | **Gripped by the rapier** | **Comments** |
| --- | --- | --- | --- | --- | --- |
| 40 Ne Viscose | 2 | Feeder 1 | Yes | Yes |  |
|  |  | Feeder 2 | Yes | Yes |  |
|  |  | Feeder 3 | Yes | Yes |  |
|  |  | Feeder 4 | Yes | Yes |  |
|  |  | Feeder 5 | Yes | Yes |  |
|  |  | Feeder 6 | Yes | Yes |  |
|  |  | Feeder 7 | Yes | Yes |  |
|  |  | Feeder 8 | Yes | Yes |  |
|  |  | Feeder 9 | Yes | Yes |  |
|  |  | Feeder 10 | Yes | Yes |  |
|  |  | Feeder 11 | Yes | Yes |  |
|  |  | Feeder 12 | Yes | No | Miss-Pick |
|  |  | Feeder 13 | Yes | Yes |  |
|  |  | Feeder 14 | Yes | Yes |  |
|  |  | Feeder 15 | Yes | Yes |  |
|  |  | Feeder 16 | Yes | Yes |  |
|  |  | Feeder 17 | Yes | Yes |  |
|  |  | Feeder 18 | Yes | Yes |  |
|  |  | Feeder 19 | Yes | Yes |  |
|  |  | Feeder 20 | Yes | Yes |  |
|  | | Number of wefts used = 20 | Number of successful weft selections =20 | Number of successful weft insertions =19 | Number of miss-picks = 1 |
| **Result for Trial 2(40 Ne Viscose)**  Weft Selection Rate for trial 2(40 Ne Viscose)  = $\frac{Number of successful selections by the Weft Selector}{Number of wefts used}$ × 100%  =$\frac{20}{20}$× 100%  =100%  Pick Insertion Rate for trial 2(40 Ne Viscose)  = $\frac{Number of successful weft insertion by the rapier}{Number of wefts used}$× 100%  =$\frac{19}{20}$× 100%  =95% | | | | | |

| **Types and count of yarn** | **Trial No.** | **Feeder No.** | **Selected by the Weft Selector** | **Gripped by the rapier** | **Comments** |
| --- | --- | --- | --- | --- | --- |
| 40 Ne Viscose | 3 | Feeder 1 | Yes | Yes |  |
|  |  | Feeder 2 | Yes | Yes |  |
|  |  | Feeder 3 | Yes | Yes |  |
|  |  | Feeder 4 | Yes | Yes |  |
|  |  | Feeder 5 | Yes | Yes |  |
|  |  | Feeder 6 | Yes | Yes |  |
|  |  | Feeder 7 | Yes | Yes |  |
|  |  | Feeder 8 | Yes | Yes |  |
|  |  | Feeder 9 | Yes | Yes |  |
|  |  | Feeder 10 | Yes | Yes |  |
|  |  | Feeder 11 | Yes | Yes |  |
|  |  | Feeder 12 | Yes | Yes |  |
|  |  | Feeder 13 | Yes | Yes |  |
|  |  | Feeder 14 | Yes | Yes |  |
|  |  | Feeder 15 | Yes | Yes |  |
|  |  | Feeder 16 | Yes | Yes |  |
|  |  | Feeder 17 | Yes | Yes |  |
|  |  | Feeder 18 | Yes | Yes |  |
|  |  | Feeder 19 | Yes | Yes |  |
|  |  | Feeder 20 | Yes | Yes |  |
|  | | Number of wefts used = 20 | Number of successful weft selections =20 | Number of successful weft insertions =20 | Number of miss-picks =0 |
| **Result for Trial 3(40 Ne Viscose)**  Weft Selection Rate for trial 3(40 Ne Viscose)  = $\frac{Number of successful selections by the Weft Selector}{Number of wefts used}$ × 100%  =$\frac{20}{20}$× 100%  =100%  Pick Insertion Rate for trial 3(40 Ne Viscose)  = $\frac{Number of successful weft insertion by the rapier}{Number of wefts used}$× 100%  =$\frac{20}{20}$× 100%  =100% | | | | | |

| **Types and count of yarn** | **Trial No.** | **Feeder No.** | **Selected by the Weft Selector** | **Gripped by the rapier** | **Comments** |
| --- | --- | --- | --- | --- | --- |
| 40 Ne Viscose | 4 | Feeder 1 | Yes | Yes |  |
|  |  | Feeder 2 | Yes | Yes |  |
|  |  | Feeder 3 | Yes | Yes |  |
|  |  | Feeder 4 | Yes | Yes |  |
|  |  | Feeder 5 | Yes | Yes |  |
|  |  | Feeder 6 | Yes | Yes |  |
|  |  | Feeder 7 | Yes | Yes |  |
|  |  | Feeder 8 | Yes | Yes |  |
|  |  | Feeder 9 | Yes | Yes |  |
|  |  | Feeder 10 | Yes | Yes |  |
|  |  | Feeder 11 | Yes | Yes |  |
|  |  | Feeder 12 | Yes | Yes |  |
|  |  | Feeder 13 | Yes | Yes |  |
|  |  | Feeder 14 | Yes | Yes |  |
|  |  | Feeder 15 | Yes | Yes |  |
|  |  | Feeder 16 | Yes | Yes |  |
|  |  | Feeder 17 | Yes | Yes |  |
|  |  | Feeder 18 | Yes | Yes |  |
|  |  | Feeder 19 | Yes | Yes |  |
|  |  | Feeder 20 | Yes | Yes |  |
|  | | Number of wefts used = 20 | Number of successful weft selections =20 | Number of successful weft insertions =20 | Number of miss-picks =0 |
| **Result for Trial 4(40 Ne Viscose)**  Weft Selection Rate for trial 4(40 Ne Viscose)  = $\frac{Number of successful selections by the Weft Selector}{Number of wefts used}$ × 100%  =$\frac{20}{20}$× 100%  =100%  Pick Insertion Rate for trial 4(40 Ne Viscose)  = $\frac{Number of successful weft insertion by the rapier}{Number of wefts used}$× 100%  =$\frac{20}{20}$× 100%  =100% | | | | | |

| **Types and count of yarn** | **Trial No.** | **Feeder No.** | **Selected by the Weft Selector** | **Gripped by the rapier** | **Comments** |
| --- | --- | --- | --- | --- | --- |
| 40 Ne Viscose | 5 | Feeder 1 | Yes | Yes |  |
|  |  | Feeder 2 | Yes | Yes |  |
|  |  | Feeder 3 | Yes | Yes |  |
|  |  | Feeder 4 | Yes | Yes |  |
|  |  | Feeder 5 | Yes | Yes |  |
|  |  | Feeder 6 | Yes | Yes |  |
|  |  | Feeder 7 | Yes | Yes |  |
|  |  | Feeder 8 | Yes | Yes |  |
|  |  | Feeder 9 | Yes | Yes |  |
|  |  | Feeder 10 | Yes | Yes |  |
|  |  | Feeder 11 | Yes | Yes |  |
|  |  | Feeder 12 | Yes | Yes |  |
|  |  | Feeder 13 | Yes | Yes |  |
|  |  | Feeder 14 | Yes | Yes |  |
|  |  | Feeder 15 | Yes | Yes |  |
|  |  | Feeder 16 | Yes | Yes |  |
|  |  | Feeder 17 | Yes | Yes |  |
|  |  | Feeder 18 | Yes | Yes |  |
|  |  | Feeder 19 | Yes | Yes |  |
|  |  | Feeder 20 | Yes | Yes |  |
|  | | Number of wefts used = 20 | Number of successful weft selections =20 | Number of successful weft insertions =20 | Number of miss-picks =0 |
| **Result for Trial 5(40 Ne Viscose)**  Weft Selection Rate for trial 5(40 Ne Viscose)  = $\frac{Number of successful selections by the Weft Selector}{Number of wefts used}$ × 100%  =$\frac{20}{20}$× 100%  =100%  Pick Insertion Rate for trial 5(40 Ne Viscose)  = $\frac{Number of successful weft insertion by the rapier}{Number of wefts used}$× 100%  =$\frac{18}{20}$× 100%  =90% | | | | | |

**Average rate of weft selection, pick insertion, and Miss-Pick for 40 Ne Viscose**

| **Trial No.** | **Weft Selection Rate** | **Pick Insertion Rate** | **Number of mis-picks** |
| --- | --- | --- | --- |
| 1 | 95% | 95% | 1 |
| 2 | 100% | 95% | 1 |
| 3 | 100% | 100% | 0 |
| 4 | 100% | 100% | 0 |
| 5 | 100% | 100% | 0 |
|  | Average rate of weft selection = 99% | Average rate of pick insertion =98% | Average Miss-Pick Rate= (2÷100) ×100%=2% |

**40 Ne Spun Polyester**

| **Types and count of yarn** | **Trial No.** | **Feeder No.** | **Selected by the Weft Selector** | **Gripped by the rapier** | **Comments** |
| --- | --- | --- | --- | --- | --- |
| 40 Ne Spun Polyester | 1 | Feeder 1 | Yes | Yes |  |
|  |  | Feeder 2 | No | No | Missed by the selector |
|  |  | Feeder 3 | Yes | Yes |  |
|  |  | Feeder 4 | Yes | Yes |  |
|  |  | Feeder 5 | Yes | Yes |  |
|  |  | Feeder 6 | Yes | Yes |  |
|  |  | Feeder 7 | Yes | Yes |  |
|  |  | Feeder 8 | Yes | Yes |  |
|  |  | Feeder 9 | Yes | Yes |  |
|  |  | Feeder 10 | Yes | Yes |  |
|  |  | Feeder 11 | Yes | Yes |  |
|  |  | Feeder 12 | Yes | Yes |  |
|  |  | Feeder 13 | Yes | Yes |  |
|  |  | Feeder 14 | Yes | Yes |  |
|  |  | Feeder 15 | Yes | Yes |  |
|  |  | Feeder 16 | Yes | Yes |  |
|  |  | Feeder 17 | Yes | Yes |  |
|  |  | Feeder 18 | Yes | Yes |  |
|  |  | Feeder 19 | Yes | Yes |  |
|  |  | Feeder 20 | Yes | Yes |  |
|  | | Number of wefts used = 20 | Number of successful weft selections =19 | Number of successful weft insertions =19 | Number of miss-picks = 1 |
| **Result for Trial 1(40 Ne Spun Polyester)**  Weft Selection Rate for trial 1(40 Ne Spun Polyester)  = $\frac{Number of successful selections by the Weft Selector}{Number of wefts used}$ × 100%  =$\frac{19}{20}$× 100%  =95%  Pick Insertion Rate for trial 1(40 Ne Spun Polyester)  = $\frac{Number of successful weft insertion by the rapier}{Number of wefts used}$× 100%  =$\frac{19}{20}$× 100%  =95% | | | | | |

| **Types and count of yarn** | **Trial No.** | **Feeder No.** | **Selected by the Weft Selector** | **Gripped by the rapier** | **Comments** |
| --- | --- | --- | --- | --- | --- |
| 40 Ne Spun Polyester | 2 | Feeder 1 | Yes | Yes |  |
|  |  | Feeder 2 | Yes | Yes |  |
|  |  | Feeder 3 | Yes | Yes |  |
|  |  | Feeder 4 | Yes | Yes |  |
|  |  | Feeder 5 | Yes | Yes |  |
|  |  | Feeder 6 | Yes | Yes |  |
|  |  | Feeder 7 | Yes | Yes |  |
|  |  | Feeder 8 | Yes | Yes |  |
|  |  | Feeder 9 | Yes | Yes |  |
|  |  | Feeder 10 | Yes | Yes |  |
|  |  | Feeder 11 | Yes | Yes |  |
|  |  | Feeder 12 | Yes | Yes |  |
|  |  | Feeder 13 | Yes | Yes |  |
|  |  | Feeder 14 | Yes | Yes |  |
|  |  | Feeder 15 | Yes | Yes |  |
|  |  | Feeder 16 | Yes | Yes |  |
|  |  | Feeder 17 | Yes | Yes |  |
|  |  | Feeder 18 | Yes | Yes |  |
|  |  | Feeder 19 | Yes | Yes |  |
|  |  | Feeder 20 | Yes | Yes |  |
|  | | Number of wefts used = 20 | Number of successful weft selections =20 | Number of successful weft insertions =20 | Number of miss-picks =0 |
| **Result for Trial 2(40 Ne Spun Polyester)**  Weft Selection Rate for trial 2(40 Ne Spun Polyester)  = $\frac{Number of successful selections by the Weft Selector}{Number of wefts used}$ × 100%  =$\frac{19}{20}$× 100%  =95%  Pick Insertion Rate for trial 2(40 Ne Spun Polyester)  = $\frac{Number of successful weft insertion by the rapier}{Number of wefts used}$× 100%  =$\frac{19}{20}$× 100%  =95% | | | | | |

| **Types and count of yarn** | **Trial No.** | **Feeder No.** | **Selected by the Weft Selector** | **Gripped by the rapier** | **Comments** |
| --- | --- | --- | --- | --- | --- |
| 40 Ne Spun Polyester | 3 | Feeder 1 | Yes | Yes |  |
|  |  | Feeder 2 | Yes | Yes |  |
|  |  | Feeder 3 | Yes | Yes |  |
|  |  | Feeder 4 | Yes | Yes |  |
|  |  | Feeder 5 | Yes | Yes |  |
|  |  | Feeder 6 | Yes | Yes |  |
|  |  | Feeder 7 | Yes | Yes |  |
|  |  | Feeder 8 | Yes | Yes |  |
|  |  | Feeder 9 | Yes | Yes |  |
|  |  | Feeder 10 | Yes | Yes |  |
|  |  | Feeder 11 | Yes | Yes |  |
|  |  | Feeder 12 | Yes | Yes |  |
|  |  | Feeder 13 | Yes | Yes |  |
|  |  | Feeder 14 | Yes | Yes |  |
|  |  | Feeder 15 | Yes | Yes |  |
|  |  | Feeder 16 | Yes | Yes |  |
|  |  | Feeder 17 | Yes | Yes |  |
|  |  | Feeder 18 | Yes | Yes |  |
|  |  | Feeder 19 | Yes | Yes |  |
|  |  | Feeder 20 | Yes | Yes |  |
|  | | Number of wefts used = 20 | Number of successful weft selections =20 | Number of successful weft insertions =20 | Number of miss-picks =0 |
| **Result for Trial 3(40 Ne Spun Polyester)**  Weft Selection Rate for trial 3(40 Ne Spun Polyester)  = $\frac{Number of successful selections by the Weft Selector}{Number of wefts used}$ × 100%  =$\frac{20}{20}$× 100%  =100%  Pick Insertion Rate for trial 3(40 Ne Spun Polyester)  = $\frac{Number of successful weft insertion by the rapier}{Number of wefts used}$× 100%  =$\frac{20}{20}$× 100%  =100% | | | | | |

| **Types and count of yarn** | **Trial No.** | **Feeder No.** | **Selected by the Weft Selector** | **Gripped by the rapier** | **Comments** |
| --- | --- | --- | --- | --- | --- |
| 40 Ne Spun Polyester | 4 | Feeder 1 | Yes | Yes |  |
|  |  | Feeder 2 | Yes | Yes |  |
|  |  | Feeder 3 | Yes | Yes |  |
|  |  | Feeder 4 | Yes | Yes |  |
|  |  | Feeder 5 | Yes | Yes |  |
|  |  | Feeder 6 | Yes | Yes |  |
|  |  | Feeder 7 | Yes | Yes |  |
|  |  | Feeder 8 | Yes | Yes |  |
|  |  | Feeder 9 | Yes | Yes |  |
|  |  | Feeder 10 | Yes | Yes |  |
|  |  | Feeder 11 | Yes | Yes |  |
|  |  | Feeder 12 | No | No | Missed by the selector |
|  |  | Feeder 13 | Yes | Yes |  |
|  |  | Feeder 14 | Yes | Yes |  |
|  |  | Feeder 15 | Yes | Yes |  |
|  |  | Feeder 16 | Yes | Yes |  |
|  |  | Feeder 17 | Yes | Yes |  |
|  |  | Feeder 18 | Yes | Yes |  |
|  |  | Feeder 19 | Yes | Yes |  |
|  |  | Feeder 20 | Yes | Yes |  |
|  | | Number of wefts used = 20 | Number of successful weft selections =19 | Number of successful weft insertions =19 | Number of miss-picks = 1 |
| **Result for Trial 4(40 Ne Spun Polyester)**  Weft Selection Rate for trial 4(40 Ne Spun Polyester)  = $\frac{Number of successful selections by the Weft Selector}{Number of wefts used}$ × 100%  =$\frac{19}{20}$× 100%  =95%  Pick Insertion Rate for trial 4(40 Ne Spun Polyester)  = $\frac{Number of successful weft insertion by the rapier}{Number of wefts used}$× 100%  =$\frac{19}{20}$× 100%  =95% | | | | | |

| **Types and count of yarn** | **Trial No.** | **Feeder No.** | **Selected by the Weft Selector** | **Gripped by the rapier** | **Comments** |
| --- | --- | --- | --- | --- | --- |
| 40 Ne Spun Polyester | 5 | Feeder 1 | Yes | Yes |  |
|  |  | Feeder 2 | Yes | Yes |  |
|  |  | Feeder 3 | Yes | Yes |  |
|  |  | Feeder 4 | Yes | Yes |  |
|  |  | Feeder 5 | Yes | Yes |  |
|  |  | Feeder 6 | Yes | Yes |  |
|  |  | Feeder 7 | Yes | Yes |  |
|  |  | Feeder 8 | Yes | Yes |  |
|  |  | Feeder 9 | Yes | Yes |  |
|  |  | Feeder 10 | Yes | Yes |  |
|  |  | Feeder 11 | Yes | Yes |  |
|  |  | Feeder 12 | Yes | Yes |  |
|  |  | Feeder 13 | Yes | Yes |  |
|  |  | Feeder 14 | Yes | Yes |  |
|  |  | Feeder 15 | Yes | Yes |  |
|  |  | Feeder 16 | Yes | Yes |  |
|  |  | Feeder 17 | Yes | Yes |  |
|  |  | Feeder 18 | Yes | Yes |  |
|  |  | Feeder 19 | Yes | Yes |  |
|  |  | Feeder 20 | Yes | Yes |  |
|  | | Number of wefts used = 20 | Number of successful weft selections =20 | Number of successful weft insertions =20 | Number of miss-picks =0 |
| **Result for Trial 5(40 Ne Spun Polyester)**  Weft Selection Rate for trial 5(40 Ne Spun Polyester)  = $\frac{Number of successful selections by the Weft Selector}{Number of wefts used}$ × 100%  =$\frac{20}{20}$× 100%  =100%  Pick Insertion Rate for trial 5(40 Ne Spun Polyester)  = $\frac{Number of successful weft insertion by the rapier}{Number of wefts used}$× 100%  =$\frac{20}{20}$× 100%  =100% | | | | | |

**Average rate of weft selection, insertion, and Miss-Pick for 40 Ne Spun Polyester**

| **Trial No.** | **Weft Selection Rate** | **Pick Insertion Rate** | **Number of mis-picks** |
| --- | --- | --- | --- |
| 1 | 95% | 95% | 1 |
| 2 | 100% | 100% | 0 |
| 3 | 100% | 100% | 0 |
| 4 | 95% | 95% | 1 |
| 5 | 100% | 100% | 0 |
|  | Average rate of weft selection = 98% | Average rate of pick insertion =98% | Average Miss-Pick Rate= (2÷100) ×100%=2% |

**Polyester 50D 96 Filaments & Lycra 40 D**

| **Types and count of yarn** | **Trial No.** | **Feeder No.** | **Selected by the Weft Selector** | **Gripped by the rapier** | **Comments** |
| --- | --- | --- | --- | --- | --- |
| Polyester 50D 96 Filaments & Lycra 40 D | 1 | Feeder 1 | Yes | Yes |  |
|  |  | Feeder 2 | Yes | Yes |  |
|  |  | Feeder 3 | Yes | Yes |  |
|  |  | Feeder 4 | Yes | Yes |  |
|  |  | Feeder 5 | Yes | Yes |  |
|  |  | Feeder 6 | Yes | Yes |  |
|  |  | Feeder 7 | Yes | Yes |  |
|  |  | Feeder 8 | No | No | Missed by the selector |
|  |  | Feeder 9 | Yes | Yes |  |
|  |  | Feeder 10 | Yes | Yes |  |
|  |  | Feeder 11 | Yes | Yes |  |
|  |  | Feeder 12 | Yes | Yes |  |
|  |  | Feeder 13 | Yes | Yes |  |
|  |  | Feeder 14 | Yes | Yes |  |
|  |  | Feeder 15 | Yes | Yes |  |
|  |  | Feeder 16 | Yes | Yes |  |
|  |  | Feeder 17 | Yes | Yes |  |
|  |  | Feeder 18 | Yes | Yes |  |
|  |  | Feeder 19 | Yes | Yes |  |
|  |  | Feeder 20 | Yes | Yes |  |
|  | | Number of wefts used = 20 | Number of successful weft selections =19 | Number of successful weft insertions =19 | Number of miss-picks =1 |
| **Result for Trial 1(Polyester 50D 96 Filaments & Lycra 40 D)**  Weft Selection Rate for trial 1(Polyester 50D 96 Filaments & Lycra 40 D)  = $\frac{Number of successful selections by the Weft Selector}{Number of wefts used}$ × 100%  =$\frac{19}{20}$× 100%  =95%  Pick Insertion Rate for trial 1(Polyester 50D 96 Filaments & Lycra 40 D)  = $\frac{Number of successful weft insertion by the rapier}{Number of wefts used}$× 100%  =$\frac{19}{20}$× 100%  =95% | | | | | |

| **Types and count of yarn** | **Trial No.** | **Feeder No.** | **Selected by the Weft Selector** | **Gripped by the rapier** | **Comments** |
| --- | --- | --- | --- | --- | --- |
| Polyester 50D 96 Filaments & Lycra 40 D | 2 | Feeder 1 | Yes | Yes |  |
|  |  | Feeder 2 | Yes | Yes |  |
|  |  | Feeder 3 | Yes | Yes |  |
|  |  | Feeder 4 | Yes | Yes |  |
|  |  | Feeder 5 | Yes | Yes |  |
|  |  | Feeder 6 | Yes | Yes |  |
|  |  | Feeder 7 | Yes | Yes |  |
|  |  | Feeder 8 | Yes | Yes |  |
|  |  | Feeder 9 | Yes | Yes |  |
|  |  | Feeder 10 | Yes | Yes |  |
|  |  | Feeder 11 | Yes | Yes |  |
|  |  | Feeder 12 | Yes | Yes |  |
|  |  | Feeder 13 | Yes | Yes |  |
|  |  | Feeder 14 | Yes | Yes |  |
|  |  | Feeder 15 | Yes | Yes |  |
|  |  | Feeder 16 | Yes | Yes |  |
|  |  | Feeder 17 | Yes | Yes |  |
|  |  | Feeder 18 | Yes | Yes |  |
|  |  | Feeder 19 | Yes | Yes |  |
|  |  | Feeder 20 | Yes | Yes |  |
|  | | Number of wefts used = 20 | Number of successful weft selections =20 | Number of successful weft insertions =20 | Number of miss-picks =0 |
| **Result for Trial 2(Polyester 50D 96 Filaments & Lycra 40 D)**  Weft Selection Rate for trial 2(Polyester 50D 96 Filaments & Lycra 40 D)  = $\frac{Number of successful selections by the Weft Selector}{Number of wefts used}$ × 100%  =$\frac{20}{20}$× 100%  =100%  Pick Insertion Rate for trial 2(Polyester 50D 96 Filaments & Lycra 40 D)  = $\frac{Number of successful weft insertion by the rapier}{Number of wefts used}$× 100%  =$\frac{20}{20}$× 100%  =100% | | | | | |

| **Types and count of yarn** | **Trial No.** | **Feeder No.** | **Selected by the Weft Selector** | **Gripped by the rapier** | **Comments** |
| --- | --- | --- | --- | --- | --- |
| Polyester 50D 96 Filaments & Lycra 40 D | 3 | Feeder 1 | Yes | Yes |  |
|  |  | Feeder 2 | Yes | Yes |  |
|  |  | Feeder 3 | Yes | Yes |  |
|  |  | Feeder 4 | Yes | Yes |  |
|  |  | Feeder 5 | Yes | Yes |  |
|  |  | Feeder 6 | Yes | Yes |  |
|  |  | Feeder 7 | Yes | Yes |  |
|  |  | Feeder 8 | Yes | Yes |  |
|  |  | Feeder 9 | Yes | Yes |  |
|  |  | Feeder 10 | Yes | Yes |  |
|  |  | Feeder 11 | Yes | Yes |  |
|  |  | Feeder 12 | Yes | Yes |  |
|  |  | Feeder 13 | Yes | Yes |  |
|  |  | Feeder 14 | Yes | Yes |  |
|  |  | Feeder 15 | Yes | Yes |  |
|  |  | Feeder 16 | Yes | Yes |  |
|  |  | Feeder 17 | Yes | Yes |  |
|  |  | Feeder 18 | Yes | Yes |  |
|  |  | Feeder 19 | Yes | Yes |  |
|  |  | Feeder 20 | Yes | Yes |  |
|  | | Number of wefts used = 20 | Number of successful weft selections =20 | Number of successful weft insertions =20 | Number of miss-picks =0 |
| **Result for Trial 3(Polyester 50D 96 Filaments & Lycra 40 D)**  Weft Selection Rate for trial 3(Polyester 50D 96 Filaments & Lycra 40 D)  = $\frac{Number of successful selections by the Weft Selector}{Number of wefts used}$ × 100%  =$\frac{20}{20}$× 100%  =100%  Pick Insertion Rate for trial 3(Polyester 50D 96 Filaments & Lycra 40 D)  = $\frac{Number of successful weft insertion by the rapier}{Number of wefts used}$× 100%  =$\frac{20}{20}$× 100%  =100% | | | | | |

| **Types and count of yarn** | **Trial No.** | **Feeder No.** | **Selected by the Weft Selector** | **Gripped by the rapier** | **Comments** |
| --- | --- | --- | --- | --- | --- |
| Polyester 50D 96 Filaments & Lycra 40 D | 4 | Feeder 1 | Yes | Yes |  |
|  |  | Feeder 2 | Yes | Yes |  |
|  |  | Feeder 3 | Yes | Yes |  |
|  |  | Feeder 4 | Yes | Yes |  |
|  |  | Feeder 5 | Yes | Yes |  |
|  |  | Feeder 6 | Yes | Yes |  |
|  |  | Feeder 7 | Yes | Yes |  |
|  |  | Feeder 8 | Yes | Yes |  |
|  |  | Feeder 9 | Yes | Yes |  |
|  |  | Feeder 10 | Yes | Yes |  |
|  |  | Feeder 11 | Yes | Yes |  |
|  |  | Feeder 12 | Yes | Yes |  |
|  |  | Feeder 13 | Yes | Yes |  |
|  |  | Feeder 14 | Yes | Yes |  |
|  |  | Feeder 15 | Yes | Yes |  |
|  |  | Feeder 16 | Yes | Yes |  |
|  |  | Feeder 17 | No | No | Missed by the selector |
|  |  | Feeder 18 | Yes | Yes |  |
|  |  | Feeder 19 | Yes | Yes |  |
|  |  | Feeder 20 | Yes | Yes |  |
|  | | Number of wefts used = 20 | Number of successful weft selections =19 | Number of successful weft insertions =19 | Number of miss-picks =1 |
| **Result for Trial 4(Polyester 50D 96 Filaments & Lycra 40 D)**  Weft Selection Rate for trial 4(Polyester 50D 96 Filaments & Lycra 40 D)  = $\frac{Number of successful selections by the Weft Selector}{Number of wefts used}$ × 100%  =$\frac{19}{20}$× 100%  =95%  Pick Insertion Rate for trial 4(Polyester 50D 96 Filaments & Lycra 40 D)  = $\frac{Number of successful weft insertion by the rapier}{Number of wefts used}$× 100%  =$\frac{19}{20}$× 100%  =95% | | | | | |

| **Types and count of yarn** | **Trial No.** | **Feeder No.** | **Selected by the Weft Selector** | **Gripped by the rapier** | **Comments** |
| --- | --- | --- | --- | --- | --- |
| Polyester 50D 96 Filaments & Lycra 40 D | 5 | Feeder 1 | Yes | Yes |  |
|  |  | Feeder 2 | Yes | Yes |  |
|  |  | Feeder 3 | Yes | Yes |  |
|  |  | Feeder 4 | Yes | Yes |  |
|  |  | Feeder 5 | Yes | Yes |  |
|  |  | Feeder 6 | Yes | Yes |  |
|  |  | Feeder 7 | Yes | Yes |  |
|  |  | Feeder 8 | Yes | Yes |  |
|  |  | Feeder 9 | Yes | Yes |  |
|  |  | Feeder 10 | Yes | Yes |  |
|  |  | Feeder 11 | Yes | Yes |  |
|  |  | Feeder 12 | Yes | Yes |  |
|  |  | Feeder 13 | Yes | Yes |  |
|  |  | Feeder 14 | Yes | Yes |  |
|  |  | Feeder 15 | Yes | Yes |  |
|  |  | Feeder 16 | Yes | Yes |  |
|  |  | Feeder 17 | Yes | Yes |  |
|  |  | Feeder 18 | Yes | Yes |  |
|  |  | Feeder 19 | Yes | Yes |  |
|  |  | Feeder 20 | Yes | Yes |  |
|  | | Number of wefts used = 20 | Number of successful weft selections =20 | Number of successful weft insertions =20 | Number of miss-picks =0 |
| **Result for Trial 5(Polyester 50D 96 Filaments & Lycra 40 D)**  Weft Selection Rate for trial 5(Polyester 50D 96 Filaments & Lycra 40 D)  = $\frac{Number of successful selections by the Weft Selector}{Number of wefts used}$ × 100%  =$\frac{20}{20}$× 100%  =100%  Pick Insertion Rate for trial 5(Polyester 50D 96 Filaments & Lycra 40 D)  = $\frac{Number of successful weft insertion by the rapier}{Number of wefts used}$× 100%  =$\frac{20}{20}$× 100%  =100% | | | | | |

**Average rate of weft selection, pick insertion and Miss-Pick for Polyester 50D 96 Filaments & Lycra 40 D**

| **Trial No.** | **Weft Selection Rate** | **Pick Insertion Rate** | **Number of mis-picks** |
| --- | --- | --- | --- |
| 1 | 95% | 95% | 1 |
| 2 | 100% | 100% | 0 |
| 3 | 100% | 100% | 0 |
| 4 | 95% | 95% | 1 |
| 5 | 100% | 100% | 0 |
|  | Average rate of weft selection = 98% | Average rate of pick insertion =98% | Average Miss-Pick Rate= (2÷100) ×100%=2% |

**16 Ne Carded Cotton**

| **Types and count of yarn** | **Trial No.** | **Feeder No.** | **Selected by the Weft Selector** | **Gripped by the rapier** | **Comments** |
| --- | --- | --- | --- | --- | --- |
| 16 Ne Carded Cotton | 1 | Feeder 1 | Yes | Yes |  |
|  |  | Feeder 2 | Yes | Yes |  |
|  |  | Feeder 3 | Yes | Yes |  |
|  |  | Feeder 4 | Yes | Yes |  |
|  |  | Feeder 5 | Yes | Yes |  |
|  |  | Feeder 6 | Yes | Yes |  |
|  |  | Feeder 7 | Yes | Yes |  |
|  |  | Feeder 8 | Yes | Yes |  |
|  |  | Feeder 9 | Yes | Yes |  |
|  |  | Feeder 10 | Yes | Yes |  |
|  |  | Feeder 11 | Yes | Yes |  |
|  |  | Feeder 12 | Yes | Yes |  |
|  |  | Feeder 13 | Yes | Yes |  |
|  |  | Feeder 14 | Yes | Yes |  |
|  |  | Feeder 15 | Yes | Yes |  |
|  |  | Feeder 16 | Yes | Yes |  |
|  |  | Feeder 17 | Yes | Yes |  |
|  |  | Feeder 18 | Yes | Yes |  |
|  |  | Feeder 19 | Yes | Yes |  |
|  |  | Feeder 20 | Yes | Yes |  |
|  | | Number of wefts used = 20 | Number of successful weft selections =20 | Number of successful weft insertions =20 | Number of miss-picks =0 |
| **Result for Trial 1(16 Ne Carded Cotton)**  Weft Selection Rate for trial 1(16 Ne Carded Cotton)  = $\frac{Number of successful selections by the Weft Selector}{Number of wefts used}$ × 100%  =$\frac{20}{20}$× 100%  =100%  Pick Insertion Rate for trial 1(16 Ne Carded Cotton)  = $\frac{Number of successful weft insertion by the rapier}{Number of wefts used}$× 100%  =$\frac{20}{20}$× 100%  =100% | | | | | |

| **Types and count of yarn** | **Trial No.** | **Feeder No.** | **Selected by the Weft Selector** | **Gripped by the rapier** | **Comments** |
| --- | --- | --- | --- | --- | --- |
| 16 Ne Carded Cotton | 2 | Feeder 1 | Yes | Yes |  |
|  |  | Feeder 2 | Yes | Yes |  |
|  |  | Feeder 3 | Yes | Yes |  |
|  |  | Feeder 4 | Yes | Yes |  |
|  |  | Feeder 5 | Yes | Yes |  |
|  |  | Feeder 6 | Yes | Yes |  |
|  |  | Feeder 7 | Yes | Yes |  |
|  |  | Feeder 8 | Yes | Yes |  |
|  |  | Feeder 9 | Yes | Yes |  |
|  |  | Feeder 10 | Yes | Yes |  |
|  |  | Feeder 11 | Yes | Yes |  |
|  |  | Feeder 12 | Yes | Yes |  |
|  |  | Feeder 13 | Yes | Yes |  |
|  |  | Feeder 14 | Yes | Yes |  |
|  |  | Feeder 15 | Yes | Yes |  |
|  |  | Feeder 16 | Yes | Yes |  |
|  |  | Feeder 17 | Yes | Yes |  |
|  |  | Feeder 18 | Yes | Yes |  |
|  |  | Feeder 19 | Yes | Yes |  |
|  |  | Feeder 20 | Yes | Yes |  |
|  | | Number of wefts used = 20 | Number of successful weft selections =20 | Number of successful weft insertions =20 | Number of miss-picks =0 |
| **Result for Trial 2(16 Ne Carded Cotton)**  Weft Selection Rate for trial 2(16 Ne Carded Cotton)  = $\frac{Number of successful selections by the Weft Selector}{Number of wefts used}$ × 100%  =$\frac{20}{20}$× 100%  =100%  Pick Insertion Rate for trial 2(16 Ne Carded Cotton)  = $\frac{Number of successful weft insertion by the rapier}{Number of wefts used}$× 100%  =$\frac{20}{20}$× 100%  =100% | | | | | |

| **Types and count of yarn** | **Trial No.** | **Feeder No.** | **Selected by the Weft Selector** | **Gripped by the rapier** | **Comments** |
| --- | --- | --- | --- | --- | --- |
| 16 Ne Carded Cotton | 3 | Feeder 1 | Yes | Yes |  |
|  |  | Feeder 2 | Yes | Yes |  |
|  |  | Feeder 3 | Yes | Yes |  |
|  |  | Feeder 4 | Yes | Yes |  |
|  |  | Feeder 5 | Yes | Yes |  |
|  |  | Feeder 6 | Yes | Yes |  |
|  |  | Feeder 7 | Yes | Yes |  |
|  |  | Feeder 8 | Yes | Yes |  |
|  |  | Feeder 9 | Yes | Yes |  |
|  |  | Feeder 10 | Yes | Yes |  |
|  |  | Feeder 11 | Yes | Yes |  |
|  |  | Feeder 12 | Yes | Yes |  |
|  |  | Feeder 13 | Yes | Yes |  |
|  |  | Feeder 14 | Yes | Yes |  |
|  |  | Feeder 15 | Yes | Yes |  |
|  |  | Feeder 16 | Yes | Yes |  |
|  |  | Feeder 17 | Yes | Yes |  |
|  |  | Feeder 18 | Yes | Yes |  |
|  |  | Feeder 19 | Yes | Yes |  |
|  |  | Feeder 20 | Yes | Yes |  |
|  | | Number of wefts used = 20 | Number of successful weft selections =20 | Number of successful weft insertions =20 | Number of miss-picks =0 |
| **Result for Trial 3(16 Ne Carded Cotton)**  Weft Selection Rate for trial 3(16 Ne Carded Cotton)  = $\frac{Number of successful selections by the Weft Selector}{Number of wefts used}$ × 100%  =$\frac{20}{20}$× 100%  =100%  Pick Insertion Rate for trial 3(16 Ne Carded Cotton)  = $\frac{Number of successful weft insertion by the rapier}{Number of wefts used}$× 100%  =$\frac{20}{20}$× 100%  =100% | | | | | |

| **Types and count of yarn** | **Trial No.** | **Feeder No.** | **Selected by the Weft Selector** | **Gripped by the rapier** | **Comments** |
| --- | --- | --- | --- | --- | --- |
| 16 Ne Carded Cotton | 4 | Feeder 1 | Yes | Yes |  |
|  |  | Feeder 2 | Yes | Yes |  |
|  |  | Feeder 3 | Yes | Yes |  |
|  |  | Feeder 4 | Yes | Yes |  |
|  |  | Feeder 5 | Yes | Yes |  |
|  |  | Feeder 6 | Yes | Yes |  |
|  |  | Feeder 7 | Yes | Yes |  |
|  |  | Feeder 8 | Yes | Yes |  |
|  |  | Feeder 9 | Yes | Yes |  |
|  |  | Feeder 10 | Yes | Yes |  |
|  |  | Feeder 11 | Yes | Yes |  |
|  |  | Feeder 12 | Yes | Yes |  |
|  |  | Feeder 13 | Yes | Yes |  |
|  |  | Feeder 14 | Yes | Yes |  |
|  |  | Feeder 15 | Yes | Yes |  |
|  |  | Feeder 16 | Yes | Yes |  |
|  |  | Feeder 17 | Yes | Yes |  |
|  |  | Feeder 18 | Yes | Yes |  |
|  |  | Feeder 19 | Yes | Yes |  |
|  |  | Feeder 20 | Yes | Yes |  |
|  | | Number of wefts used = 20 | Number of successful weft selections =20 | Number of successful weft insertions =20 | Number of miss-picks =0 |
| **Result for Trial 4(16 Ne Carded Cotton)**  Weft Selection Rate for trial 4(16 Ne Carded Cotton)  = $\frac{Number of successful selections by the Weft Selector}{Number of wefts used}$ × 100%  =$\frac{20}{20}$× 100%  =100%  Pick Insertion Rate for trial 4(16 Ne Carded Cotton)  = $\frac{Number of successful weft insertion by the rapier}{Number of wefts used}$× 100%  =$\frac{20}{20}$× 100%  =100% | | | | | |

| **Types and count of yarn** | **Trial No.** | **Feeder No.** | **Selected by the Weft Selector** | **Gripped by the rapier** | **Comments** |
| --- | --- | --- | --- | --- | --- |
| 16 Ne Carded Cotton | 5 | Feeder 1 | Yes | Yes |  |
|  |  | Feeder 2 | Yes | Yes |  |
|  |  | Feeder 3 | Yes | Yes |  |
|  |  | Feeder 4 | Yes | Yes |  |
|  |  | Feeder 5 | Yes | Yes |  |
|  |  | Feeder 6 | Yes | Yes |  |
|  |  | Feeder 7 | Yes | Yes |  |
|  |  | Feeder 8 | Yes | Yes |  |
|  |  | Feeder 9 | Yes | Yes |  |
|  |  | Feeder 10 | Yes | Yes |  |
|  |  | Feeder 11 | Yes | Yes |  |
|  |  | Feeder 12 | Yes | Yes |  |
|  |  | Feeder 13 | Yes | Yes |  |
|  |  | Feeder 14 | Yes | Yes |  |
|  |  | Feeder 15 | Yes | Yes |  |
|  |  | Feeder 16 | Yes | Yes |  |
|  |  | Feeder 17 | Yes | Yes |  |
|  |  | Feeder 18 | Yes | Yes |  |
|  |  | Feeder 19 | Yes | Yes |  |
|  |  | Feeder 20 | Yes | Yes |  |
|  | | Number of wefts used = 20 | Number of successful weft selections =20 | Number of successful weft insertions =20 | Number of miss-picks =0 |
| **Result for Trial 5(16 Ne Carded Cotton)**  Weft Selection Rate for trial 5(16 Ne Carded Cotton)  = $\frac{Number of successful selections by the Weft Selector}{Number of wefts used}$ × 100%  =$\frac{20}{20}$× 100%  =100%  Pick Insertion Rate for trial 5(16 Ne Carded Cotton)  = $\frac{Number of successful weft insertion by the rapier}{Number of wefts used}$× 100%  =$\frac{20}{20}$× 100%  =100% | | | | | |

**Average rate of weft selection, pick insertion, and Miss-Pick for 16 Ne Carded Cotton**

| **Trial No.** | **Weft Selection Rate** | **Pick Insertion Rate** | **Number of mis-picks** |
| --- | --- | --- | --- |
| 1 | 100% | 100% | 0 |
| 2 | 100% | 100% | 0 |
| 3 | 100% | 100% | 0 |
| 4 | 100% | 100% | 0 |
| 5 | 100% | 100% | 0 |
|  | Average rate of weft selection = 100% | Average rate of pick insertion =100% | Average Miss-Pick Rate= (0÷100) ×100%=0% |

**10 Ne Carded Cotton**

| **Types and count of yarn** | **Trial No.** | **Feeder No.** | **Selected by the Weft Selector** | **Gripped by the rapier** | **Comments** |
| --- | --- | --- | --- | --- | --- |
| 10 Ne Carded Cotton | 1 | Feeder 1 | Yes | Yes |  |
|  |  | Feeder 2 | Yes | Yes |  |
|  |  | Feeder 3 | Yes | Yes |  |
|  |  | Feeder 4 | Yes | Yes |  |
|  |  | Feeder 5 | Yes | Yes |  |
|  |  | Feeder 6 | Yes | Yes |  |
|  |  | Feeder 7 | Yes | Yes |  |
|  |  | Feeder 8 | Yes | Yes |  |
|  |  | Feeder 9 | Yes | Yes |  |
|  |  | Feeder 10 | Yes | Yes |  |
|  |  | Feeder 11 | Yes | Yes |  |
|  |  | Feeder 12 | Yes | Yes |  |
|  |  | Feeder 13 | Yes | Yes |  |
|  |  | Feeder 14 | Yes | Yes |  |
|  |  | Feeder 15 | Yes | Yes |  |
|  |  | Feeder 16 | Yes | Yes |  |
|  |  | Feeder 17 | Yes | Yes |  |
|  |  | Feeder 18 | Yes | Yes |  |
|  |  | Feeder 19 | Yes | Yes |  |
|  |  | Feeder 20 | Yes | Yes |  |
|  | | Number of wefts used = 20 | Number of successful weft selections =20 | Number of successful weft insertions =20 | Number of miss-picks =0 |
| **Result for Trial 1(10 Ne Carded Cotton)**  Weft Selection Rate for trial 1(10 Ne Carded Cotton)  = $\frac{Number of successful selections by the Weft Selector}{Number of wefts used}$ × 100%  =$\frac{20}{20}$× 100%  =100%  Pick Insertion Rate for trial 1(10 Ne Carded Cotton)  = $\frac{Number of successful weft insertion by the rapier}{Number of wefts used}$× 100%  =$\frac{20}{20}$× 100%  =100% | | | | | |

| **Types and count of yarn** | **Trial No.** | **Feeder No.** | **Selected by the Weft Selector** | **Gripped by the rapier** | **Comments** |
| --- | --- | --- | --- | --- | --- |
| 10 Ne Carded Cotton | 2 | Feeder 1 | Yes | Yes |  |
|  |  | Feeder 2 | Yes | Yes |  |
|  |  | Feeder 3 | Yes | Yes |  |
|  |  | Feeder 4 | Yes | Yes |  |
|  |  | Feeder 5 | Yes | Yes |  |
|  |  | Feeder 6 | Yes | Yes |  |
|  |  | Feeder 7 | Yes | Yes |  |
|  |  | Feeder 8 | Yes | Yes |  |
|  |  | Feeder 9 | Yes | Yes |  |
|  |  | Feeder 10 | Yes | Yes |  |
|  |  | Feeder 11 | Yes | Yes |  |
|  |  | Feeder 12 | Yes | Yes |  |
|  |  | Feeder 13 | Yes | Yes |  |
|  |  | Feeder 14 | Yes | Yes |  |
|  |  | Feeder 15 | Yes | Yes |  |
|  |  | Feeder 16 | Yes | Yes |  |
|  |  | Feeder 17 | Yes | Yes |  |
|  |  | Feeder 18 | Yes | Yes |  |
|  |  | Feeder 19 | Yes | Yes |  |
|  |  | Feeder 20 | Yes | Yes |  |
|  | | Number of wefts used = 20 | Number of successful weft selections =20 | Number of successful weft insertions =20 | Number of miss-picks =0 |
| **Result for Trial 2(10 Ne Carded Cotton)**  Weft Selection Rate for trial 2(10 Ne Carded Cotton)  = $\frac{Number of successful selections by the Weft Selector}{Number of wefts used}$ × 100%  =$\frac{20}{20}$× 100%  =100%  Pick Insertion Rate for trial 2(10 Ne Carded Cotton)  = $\frac{Number of successful weft insertion by the rapier}{Number of wefts used}$× 100%  =$\frac{20}{20}$× 100%  =100% | | | | | |

| **Types and count of yarn** | **Trial No.** | **Feeder No.** | **Selected by the Weft Selector** | **Gripped by the rapier** | **Comments** |
| --- | --- | --- | --- | --- | --- |
| 10 Ne Carded Cotton | 3 | Feeder 1 | Yes | Yes |  |
|  |  | Feeder 2 | Yes | Yes |  |
|  |  | Feeder 3 | Yes | Yes |  |
|  |  | Feeder 4 | Yes | Yes |  |
|  |  | Feeder 5 | Yes | Yes |  |
|  |  | Feeder 6 | Yes | Yes |  |
|  |  | Feeder 7 | Yes | Yes |  |
|  |  | Feeder 8 | Yes | Yes |  |
|  |  | Feeder 9 | Yes | Yes |  |
|  |  | Feeder 10 | Yes | Yes |  |
|  |  | Feeder 11 | Yes | Yes |  |
|  |  | Feeder 12 | Yes | Yes |  |
|  |  | Feeder 13 | Yes | Yes |  |
|  |  | Feeder 14 | Yes | Yes |  |
|  |  | Feeder 15 | Yes | Yes |  |
|  |  | Feeder 16 | Yes | Yes |  |
|  |  | Feeder 17 | Yes | Yes |  |
|  |  | Feeder 18 | Yes | Yes |  |
|  |  | Feeder 19 | Yes | Yes |  |
|  |  | Feeder 20 | Yes | Yes |  |
|  | | Number of wefts used = 20 | Number of successful weft selections =20 | Number of successful weft insertions =20 | Number of miss-picks =0 |
| **Result for Trial 3(10 Ne Carded Cotton)**  Weft Selection Rate for trial 3(10 Ne Carded Cotton)  = $\frac{Number of successful selections by the Weft Selector}{Number of wefts used}$ × 100%  =$\frac{20}{20}$× 100%  =100%  Pick Insertion Rate for trial 3(10 Ne Carded Cotton)  = $\frac{Number of successful weft insertion by the rapier}{Number of wefts used}$× 100%  =$\frac{20}{20}$× 100%  =100% | | | | | |

| **Types and count of yarn** | **Trial No.** | **Feeder No.** | **Selected by the Weft Selector** | **Gripped by the rapier** | **Comments** |
| --- | --- | --- | --- | --- | --- |
| 10 Ne Carded Cotton | 4 | Feeder 1 | Yes | Yes |  |
|  |  | Feeder 2 | Yes | Yes |  |
|  |  | Feeder 3 | Yes | Yes |  |
|  |  | Feeder 4 | Yes | Yes |  |
|  |  | Feeder 5 | Yes | Yes |  |
|  |  | Feeder 6 | Yes | Yes |  |
|  |  | Feeder 7 | Yes | Yes |  |
|  |  | Feeder 8 | Yes | Yes |  |
|  |  | Feeder 9 | Yes | Yes |  |
|  |  | Feeder 10 | Yes | Yes |  |
|  |  | Feeder 11 | Yes | Yes |  |
|  |  | Feeder 12 | Yes | Yes |  |
|  |  | Feeder 13 | Yes | Yes |  |
|  |  | Feeder 14 | Yes | Yes |  |
|  |  | Feeder 15 | Yes | Yes |  |
|  |  | Feeder 16 | Yes | Yes |  |
|  |  | Feeder 17 | Yes | Yes |  |
|  |  | Feeder 18 | Yes | Yes |  |
|  |  | Feeder 19 | Yes | Yes |  |
|  |  | Feeder 20 | Yes | Yes |  |
|  | | Number of wefts used = 20 | Number of successful weft selections =20 | Number of successful weft insertions =20 | Number of miss-picks =0 |
| **Result for Trial 4(10 Ne Carded Cotton)**  Weft Selection Rate for trial 4(10 Ne Carded Cotton)  = $\frac{Number of successful selections by the Weft Selector}{Number of wefts used}$ × 100%  =$\frac{20}{20}$× 100%  =100%  Pick Insertion Rate for trial 4(10 Ne Carded Cotton)  = $\frac{Number of successful weft insertion by the rapier}{Number of wefts used}$× 100%  =$\frac{20}{20}$× 100%  =100% | | | | | |

| **Types and count of yarn** | **Trial No.** | **Feeder No.** | **Selected by the Weft Selector** | **Gripped by the rapier** | **Comments** |
| --- | --- | --- | --- | --- | --- |
| 10 Ne Carded Cotton | 5 | Feeder 1 | Yes | Yes |  |
|  |  | Feeder 2 | Yes | Yes |  |
|  |  | Feeder 3 | Yes | Yes |  |
|  |  | Feeder 4 | Yes | Yes |  |
|  |  | Feeder 5 | Yes | Yes |  |
|  |  | Feeder 6 | Yes | Yes |  |
|  |  | Feeder 7 | Yes | Yes |  |
|  |  | Feeder 8 | Yes | Yes |  |
|  |  | Feeder 9 | Yes | Yes |  |
|  |  | Feeder 10 | Yes | Yes |  |
|  |  | Feeder 11 | Yes | Yes |  |
|  |  | Feeder 12 | Yes | Yes |  |
|  |  | Feeder 13 | Yes | Yes |  |
|  |  | Feeder 14 | Yes | Yes |  |
|  |  | Feeder 15 | Yes | Yes |  |
|  |  | Feeder 16 | Yes | Yes |  |
|  |  | Feeder 17 | Yes | Yes |  |
|  |  | Feeder 18 | Yes | Yes |  |
|  |  | Feeder 19 | Yes | Yes |  |
|  |  | Feeder 20 | Yes | Yes |  |
|  | | Number of wefts used = 20 | Number of successful weft selections =20 | Number of successful weft insertions =20 | Number of miss-picks =0 |
| **Result for Trial 5(10 Ne Carded Cotton)**  Weft Selection Rate for trial 5(10 Ne Carded Cotton)  = $\frac{Number of successful selections by the Weft Selector}{Number of wefts used}$ × 100%  =$\frac{20}{20}$× 100%  =100%  Pick Insertion Rate for trial 5(10 Ne Carded Cotton)  = $\frac{Number of successful weft insertion by the rapier}{Number of wefts used}$× 100%  =$\frac{20}{20}$× 100%  =100% | | | | | |

**Average rate of weft selection, pick insertion, and Miss-Pick for 10 Ne Carded Cotton**

| **Trial No.** | **Weft Selection Rate** | **Pick Insertion Rate** | **Number of mis-picks** |
| --- | --- | --- | --- |
| 1 | 100% | 100% | 0 |
| 2 | 100% | 100% | 0 |
| 3 | 100% | 100% | 0 |
| 4 | 100% | 100% | 0 |
| 5 | 100% | 100% | 0 |
|  | Average rate of weft selection = 100% | Average rate of pick insertion =100% | Average Miss-Pick Rate= (0÷100)×100%=0% |

**20 Ne Carded Cotton**

| **Types and count of yarn** | **Trial No.** | **Feeder No.** | **Selected by the Weft Selector** | **Gripped by the rapier** | **Comments** |
| --- | --- | --- | --- | --- | --- |
| 20 Ne Carded Cotton | 1 | Feeder 1 | Yes | Yes |  |
|  |  | Feeder 2 | Yes | Yes |  |
|  |  | Feeder 3 | Yes | Yes |  |
|  |  | Feeder 4 | Yes | Yes |  |
|  |  | Feeder 5 | Yes | Yes |  |
|  |  | Feeder 6 | Yes | Yes |  |
|  |  | Feeder 7 | Yes | Yes |  |
|  |  | Feeder 8 | Yes | Yes |  |
|  |  | Feeder 9 | Yes | Yes |  |
|  |  | Feeder 10 | Yes | Yes |  |
|  |  | Feeder 11 | Yes | Yes |  |
|  |  | Feeder 12 | Yes | Yes |  |
|  |  | Feeder 13 | Yes | Yes |  |
|  |  | Feeder 14 | Yes | Yes |  |
|  |  | Feeder 15 | Yes | Yes |  |
|  |  | Feeder 16 | Yes | Yes |  |
|  |  | Feeder 17 | Yes | Yes |  |
|  |  | Feeder 18 | Yes | Yes |  |
|  |  | Feeder 19 | Yes | Yes |  |
|  |  | Feeder 20 | Yes | Yes |  |
|  | | Number of wefts used = 20 | Number of successful weft selections =20 | Number of successful weft insertions =20 | Number of miss-picks =0 |
| **Result for Trial 1(20 Ne Carded Cotton)**  Weft Selection Rate for trial 1(20 Ne Carded Cotton)  = $\frac{Number of successful selections by the Weft Selector}{Number of wefts used}$ × 100%  =$\frac{20}{20}$× 100%  =100%  Pick Insertion Rate for trial 1(20 Ne Carded Cotton)  = $\frac{Number of successful weft insertion by the rapier}{Number of wefts used}$× 100%  =$\frac{20}{20}$× 100%  =100% | | | | | |

| **Types and count of yarn** | **Trial No.** | **Feeder No.** | **Selected by the Weft Selector** | **Gripped by the rapier** | **Comments** |
| --- | --- | --- | --- | --- | --- |
| 20 Ne Carded Cotton | 2 | Feeder 1 | Yes | Yes |  |
|  |  | Feeder 2 | Yes | Yes |  |
|  |  | Feeder 3 | Yes | Yes |  |
|  |  | Feeder 4 | Yes | Yes |  |
|  |  | Feeder 5 | Yes | Yes |  |
|  |  | Feeder 6 | Yes | Yes |  |
|  |  | Feeder 7 | Yes | Yes |  |
|  |  | Feeder 8 | Yes | Yes |  |
|  |  | Feeder 9 | Yes | Yes |  |
|  |  | Feeder 10 | Yes | Yes |  |
|  |  | Feeder 11 | Yes | Yes |  |
|  |  | Feeder 12 | Yes | Yes |  |
|  |  | Feeder 13 | Yes | Yes |  |
|  |  | Feeder 14 | Yes | Yes |  |
|  |  | Feeder 15 | Yes | Yes |  |
|  |  | Feeder 16 | Yes | Yes |  |
|  |  | Feeder 17 | Yes | Yes |  |
|  |  | Feeder 18 | Yes | Yes |  |
|  |  | Feeder 19 | Yes | Yes |  |
|  |  | Feeder 20 | Yes | Yes |  |
|  | | Number of wefts used = 20 | Number of successful weft selections =20 | Number of successful weft insertions =20 | Number of miss-picks =0 |
| **Result for Trial 2(20 Ne Carded Cotton)**  Weft Selection Rate for trial 2(20 Ne Carded Cotton)  = $\frac{Number of successful selections by the Weft Selector}{Number of wefts used}$ × 100%  =$\frac{20}{20}$× 100%  =100%  Pick Insertion Rate for trial 2(20 Ne Carded Cotton)  = $\frac{Number of successful weft insertion by the rapier}{Number of wefts used}$× 100%  =$\frac{20}{20}$× 100%  =100% | | | | | |

| **Types and count of yarn** | **Trial No.** | **Feeder No.** | **Selected by the Weft Selector** | **Gripped by the rapier** | **Comments** |
| --- | --- | --- | --- | --- | --- |
| 20 Ne Carded Cotton | 3 | Feeder 1 | Yes | Yes |  |
|  |  | Feeder 2 | Yes | Yes |  |
|  |  | Feeder 3 | Yes | Yes |  |
|  |  | Feeder 4 | Yes | Yes |  |
|  |  | Feeder 5 | Yes | Yes |  |
|  |  | Feeder 6 | Yes | Yes |  |
|  |  | Feeder 7 | Yes | Yes |  |
|  |  | Feeder 8 | Yes | Yes |  |
|  |  | Feeder 9 | Yes | Yes |  |
|  |  | Feeder 10 | Yes | Yes |  |
|  |  | Feeder 11 | Yes | Yes |  |
|  |  | Feeder 12 | Yes | Yes |  |
|  |  | Feeder 13 | Yes | Yes |  |
|  |  | Feeder 14 | Yes | Yes |  |
|  |  | Feeder 15 | Yes | Yes |  |
|  |  | Feeder 16 | Yes | Yes |  |
|  |  | Feeder 17 | Yes | Yes |  |
|  |  | Feeder 18 | Yes | Yes |  |
|  |  | Feeder 19 | Yes | Yes |  |
|  |  | Feeder 20 | Yes | Yes |  |
|  | | Number of wefts used = 20 | Number of successful weft selections =20 | Number of successful weft insertions =20 | Number of miss-picks =0 |
| **Result for Trial 3(20 Ne Carded Cotton)**  Weft Selection Rate for trial 3(20 Ne Carded Cotton)  = $\frac{Number of successful selections by the Weft Selector}{Number of wefts used}$ × 100%  =$\frac{20}{20}$× 100%  =100%  Pick Insertion Rate for trial 3(20 Ne Carded Cotton)  = $\frac{Number of successful weft insertion by the rapier}{Number of wefts used}$× 100%  =$\frac{20}{20}$× 100%  =100% | | | | | |

| **Types and count of yarn** | **Trial No.** | **Feeder No.** | **Selected by the Weft Selector** | **Gripped by the rapier** | **Comments** |
| --- | --- | --- | --- | --- | --- |
| 20 Ne Carded Cotton | 4 | Feeder 1 | Yes | Yes |  |
|  |  | Feeder 2 | Yes | Yes |  |
|  |  | Feeder 3 | Yes | Yes |  |
|  |  | Feeder 4 | Yes | Yes |  |
|  |  | Feeder 5 | Yes | Yes |  |
|  |  | Feeder 6 | Yes | Yes |  |
|  |  | Feeder 7 | Yes | Yes |  |
|  |  | Feeder 8 | Yes | Yes |  |
|  |  | Feeder 9 | Yes | Yes |  |
|  |  | Feeder 10 | Yes | Yes |  |
|  |  | Feeder 11 | Yes | Yes |  |
|  |  | Feeder 12 | Yes | Yes |  |
|  |  | Feeder 13 | Yes | Yes |  |
|  |  | Feeder 14 | Yes | Yes |  |
|  |  | Feeder 15 | Yes | Yes |  |
|  |  | Feeder 16 | Yes | Yes |  |
|  |  | Feeder 17 | Yes | Yes |  |
|  |  | Feeder 18 | Yes | Yes |  |
|  |  | Feeder 19 | Yes | Yes |  |
|  |  | Feeder 20 | Yes | Yes |  |
|  | | Number of wefts used = 20 | Number of successful weft selections =20 | Number of successful weft insertions =20 | Number of miss-picks =0 |
| **Result for Trial 4(20 Ne Carded Cotton)**  Weft Selection Rate for trial 4(20 Ne Carded Cotton)  = $\frac{Number of successful selections by the Weft Selector}{Number of wefts used}$ × 100%  =$\frac{20}{20}$× 100%  =100%  Pick Insertion Rate for trial 4(20 Ne Carded Cotton)  = $\frac{Number of successful weft insertion by the rapier}{Number of wefts used}$× 100%  =$\frac{20}{20}$× 100%  =100% | | | | | |

| **Types and count of yarn** | **Trial No.** | **Feeder No.** | **Selected by the Weft Selector** | **Gripped by the rapier** | **Comments** |
| --- | --- | --- | --- | --- | --- |
| 20 Ne Carded Cotton | 5 | Feeder 1 | Yes | Yes |  |
|  |  | Feeder 2 | Yes | Yes |  |
|  |  | Feeder 3 | Yes | Yes |  |
|  |  | Feeder 4 | Yes | Yes |  |
|  |  | Feeder 5 | Yes | Yes |  |
|  |  | Feeder 6 | Yes | Yes |  |
|  |  | Feeder 7 | Yes | Yes |  |
|  |  | Feeder 8 | Yes | Yes |  |
|  |  | Feeder 9 | Yes | Yes |  |
|  |  | Feeder 10 | Yes | Yes |  |
|  |  | Feeder 11 | Yes | Yes |  |
|  |  | Feeder 12 | Yes | Yes |  |
|  |  | Feeder 13 | Yes | Yes |  |
|  |  | Feeder 14 | Yes | Yes |  |
|  |  | Feeder 15 | Yes | Yes |  |
|  |  | Feeder 16 | Yes | Yes |  |
|  |  | Feeder 17 | Yes | Yes |  |
|  |  | Feeder 18 | Yes | Yes |  |
|  |  | Feeder 19 | Yes | Yes |  |
|  |  | Feeder 20 | Yes | Yes |  |
|  | | Number of wefts used = 20 | Number of successful weft selections =20 | Number of successful weft insertions =20 | Number of miss-picks =0 |
| **Result for Trial 5(20 Ne Carded Cotton)**  Weft Selection Rate for trial 5(20 Ne Carded Cotton)  = $\frac{Number of successful selections by the Weft Selector}{Number of wefts used}$ × 100%  =$\frac{20}{20}$× 100%  =100%  Pick Insertion Rate for trial 5(20 Ne Carded Cotton)  = $\frac{Number of successful weft insertion by the rapier}{Number of wefts used}$× 100%  =$\frac{20}{20}$× 100%  =100% | | | | | |

**Average rate of weft selection, pick insertion, and Miss-Pick for 20 Ne Carded Cotton**

| **Trial No.** | **Weft Selection Rate** | **Pick Insertion Rate** | **Number of mis-picks** |
| --- | --- | --- | --- |
| 1 | 100% | 100% | 0 |
| 2 | 100% | 100% | 0 |
| 3 | 100% | 100% | 0 |
| 4 | 100% | 100% | 0 |
| 5 | 100% | 100% | 0 |
|  | Average rate of weft selection = 100% | Average rate of pick insertion =100% | Average Miss-Pick Rate= (0÷100) ×100%=0% |

**Overall Weft selection, Pick insertion, and Miss-Pick rate**

| **Type of yarn** | **Weft Selection Rate** | **Pick Insertion Rate** | **Miss-pick rate** |
| --- | --- | --- | --- |
| 40 Ne Carded Cotton | 99% | 99% | 1% |
| 6 Ne Carded Cotton | 100% | 100% | 0% |
| 20 Ne Open-end | 100% | 100% | 0% |
| 20 Ne Viscose | 100% | 100% | 0% |
| 30 Ne Viscose | 99% | 99% | 1% |
| 40 Ne Viscose | 99% | 98% | 2% |
| 40 Ne spun polyester | 98% | 98% | 2% |
| Polyester 50D 96 Filaments & Lycra  40 D | 98% | 98% | 2% |
| 16 Ne carded cotton | 100% | 100% | 0% |
| 10 Ne carded cotton | 100% | 100% | 0% |
| 20 Ne carded cotton | 100% | 100% | 0% |
|  | Average rate of weft selection =99.36% | Average rate of pick insertion =99.27% | Average Miss-Pick Rate =0.73 % |
