# Supplementary material for: Development of a mechatronic weft selector to enhance patterning capacity in Rapier looms
Source: PLoS One. 2025 Dec 9;20(12):e0338603. doi: 10.1371/journal.pone.0338603 (PMC12688087; doi:10.1371/journal.pone.0338603)
Supplement: Attachment — Submitted filename: Response to the Reviewer.docx [file pone.0338603.s007.docx]

**Response to the Reviewers**

The authors are grateful to the reviewers for their valuable comments. The manuscript has been revised in accordance with the provided advice. The correction has been highlighted in red in the revised version of the manuscript with track changes.

**Reviewer 1:**

1. **Title:**
   • The current title is informal and includes redundant phrasing. Suggested revision:
   "Development of a Mechatronic Weft Selector to Enhance Patterning Capacity in Rapier Looms"

Author’s reply: Necessary correction has been made according to the suggestion.

2. **Abstract:**
   • Add quantitative results (e.g., number of trials, speed, types of yarns).

Author’s reply: The above-mentioned quantitative results have been added to the Abstract, and also a supplementary document titled **“Weft Selection and Insertion Rate” (S2 Appendix)** has been included, providing detailed information on the yarn types used and the corresponding number of trials conducted.

• Avoid vague novelty claims unless supported by comparative literature.

Author’s reply: Owing to the commercial nature of loom manufacturing, limited data are available in the form of published research articles. Therefore, relevant research papers, patents, and technical brochures were thoroughly reviewed to identify existing weft selection capacities, which, to the best of our knowledge, reach up to 16. Based on this analysis, the proposed system introduces a redesigned weft selection mechanism that extends this capacity to 20, thereby demonstrating a measurable improvement over the current state of the art.

3. **Introduction:**

• The literature review should include more peer-reviewed technical papers rather than brochures.

Author’s reply: Necessary correction has been made according to the suggestion. Brochures with lower weft selection capacity have been removed (Previous reference no. 3, 4, 5, 7, 10, 12, 13), and new research articles have been added.

• Clearly state the research objectives and novel contributions at the end of the section.

Author’s reply: Necessary correction has been made according to the suggestion.

4. **Design and Methods:**

• Improve scientific framing of design descriptions (currently too procedural).

Author’s reply: Necessary correction has been made according to the suggestion.

• Embed all referenced figures.

Author’s reply: Necessary correction has been made according to the suggestion.

• Add performance metrics (actuation timing, power consumption, durability).

Author’s reply: Information regarding durability, power consumption, and electrical performance analysis has been added to the “Control Unit Development” section.

And, the “Operational Methodology” contains the parameters such as actuation timing, picks per minute.

• Consider including CAD diagrams or exploded views of the module.

Author’s reply: Diagrams created using **Adobe Illustrator** have already been included in the submitted manuscript

5. **Operational Methodology:**
• Add visuals such as flowcharts or timing diagrams.

Author’s reply: The sequence of the steps in weft selection has already been discussed in the operational methodology.

• Move TINKERCAD simulation details to supplementary materials.

Author’s reply: It has been moved to supplementary materials (S1 Appendix).

6. **Results and Evaluation:**
• Benchmark your system against existing commercial devices (DORNIER, ITEMA, PICANOL).

Author’s reply: The primary objective of this research was to present a novel design, develop a functional prototype with improved weft selection capacity, and validate its operational feasibility. As the work represents a prototype-level development, it is not yet at a stage suitable for direct benchmarking against fully commercialized systems such as DORNIER, ITEMA, or PICANOL.

• Include more performance metrics: insertion time, miss-pick rate, energy use.

Author’s reply: Miss-pick rate has been discussed in the “**Discussion and Implications**” Section. And electrical performance analysis has been added to the “Control Unit Development” section. The device integration with the loom will be done as a future extension of this work. So, the weft insertion time, related data, is not available at present. The rapier is added as a prototype here to demonstrate the yarn gripping only.

7. **Discussion:**
• Excellent recognition of the rotor's role in angular stabilization.

Author’s reply: The authors appreciate the reviewer’s positive observation regarding the rotor’s contribution to angular stabilization.

• Add commentary on system scalability and industrial deployment potential.

Author’s reply: Additional discussion has been included on system scalability and the potential of industrial integration. The revised manuscript describes how this system can be integrated with the existing weaving machines and outlines how it can be scaled for broader applications.

8. **Conclusion:**
• Reiterate key performance outcomes.

Author’s reply: Operational speed, weft selection rate, pick insertion rate, miss pick rate, power requirements, and energy consumption have been discussed in the “Conclusion” section.

• Clarify next steps in industrial integration and synchronization with real loom cycles.

Author’s reply: The steps have been discussed in the above-mentioned section. After the loom integration, synchronization can be done as a further extension of this work.

9. **References:**
• Include more peer-reviewed references to increase academic credibility.

Author’s reply: Additional peer-reviewed references have been incorporated into the revised manuscript to enhance its academic credibility. Newly added references are reference no. 9, 11, 12, 19, 21, 24

**Reviewer 2:**

(1) How was the claimed 100% selection success verified over long production runs?

Author’s reply: A detailed report has been provided as a supplementary document titled **“Weft Selection and Insertion Rate” (See S2 Appendix).** It was prepared by conducting a test on 11 different yarns, each subjected to 5 trials using 20 feeders. A total of 1100 yarns were selected. This test provided more precise data on the weft selection rate (99.36%), pick insertion rate (99.27%), which are near 100%.

(2) Is there data on operational speed and any effect on loom throughput?

Author’s reply: The “Operational Methodology” contains the parameters such as operational speed, denoted as PPM (picks per minute), and actuation time. This device was designed as a prototype to show the design and its workability. The future development will be done on the loom integration, and the effect of the operational speed will be evaluated.

(3) Were yarn breakages or mis-picks quantified across yarn types?

Author’s reply: A supplementary document titled **“Weft Selection and Insertion Rate” (See S2 Appendix),** contains the miss-pick, which was 0.73%. 1100 yarns were taken to produce the test result.

(4) How stable is the angle control when the feeder spacing is further reduced?

Author’s reply: The algorithm can determine the feeders' angular position from the given weft pattern, and from this data, it synchronizes the module’s angular position, which is precisely controlled and held in position by the stepper motor. This motor can rotate 0.9 degrees/ step precisely.

(5) Why were no industrial weft selection benchmarks used for comparison?

Author’s reply: The primary objective of this research was to present a novel design, develop a functional prototype with improved weft selection capacity, and validate its operational feasibility. As the work represents a prototype-level development, it is not yet at a stage suitable for direct benchmarking against fully commercialized systems.

(6) Could pneumatic air pressure variation impact yarn quality over time?

Author’s reply: The maximum air pressure (1.27 bar ) in the circular tube is much less than that used in the Air jet looms (2.5 to 5 bar), and this air supply is provided to 20 feeders. Moreover, the device allows the air flow for a very short period of time during the weft catching, and only 3 inches of the yarn is subjected to air flow. So, it will not affect the yarn quality largely.

(7) How is the Arduino control synchronized with actual loom cycles?

Author’s reply: This will be done as future development, and its planning has been discussed in the “Discussion and Implication” section.

(8) Are there durability tests showing wearing of moving parts and guides?

Author’s reply: Though we tried to make the system with high-quality components but the durability largely depends on its electronic components, which have been discussed in the “Control Unit Development” section.

(9) What is the cost implication versus existing multi-selector systems?

Author’s reply: This device was developed as a prototype. It is not at the stage to be compared with commercial ones.

(10) Could vibration or electrical noise affect controller accuracy in real looms?

Author’s reply: This will be evaluated as part of the future development and Loom. Integration.
